# Top-down corticostriatal control of adaptive restraint during motivational conflict

**DOI:** 10.1126/sciadv.aeg2327

**Published:** 2026-09-11

**Authors:** Elizabeth Illescas-Huerta, Eduardo Hernández-Ortiz, Francisco Sotres-Bayon

**Affiliations:** Neuroscience Division, Institute of Cell Physiology, National Autonomous University of Mexico, Mexico City 04510, Mexico.

## Abstract

Adaptive behavior under threat requires withholding reward pursuit when past experiences predict danger. Here, we identified a corticostriatal circuit that enables behavioral restraint during motivational conflict. Using a seminaturalistic foraging task in male rats, we found that inactivation of the prelimbic cortex (PL) abolished restraint without impairing memory, and single-unit recordings revealed transient PL firing increases at decision points in conflict trials. c-Fos mapping showed selective engagement of the PL and nucleus accumbens core (NAcC) but not the nucleus accumbens shell. Inactivation further revealed that the anterior NAcC (aNAcC), but not the posterior NAcC, is necessary for restraint as posterior disruption broadly reduced reward-seeking regardless of threat. Silencing PL→aNAcC projections reproduced aNAcC effects without affecting memory or motivation, and this pathway was required under learned, but not innate, threats. Together, these findings identify a corticostriatal pathway through which PL control of aNAcC supports learned threat–guided restraint during reward seeking, revealing a top-down circuit architecture for adaptive action control during motivational conflict.

## INTRODUCTION

Survival in dynamic environments requires urgent decisions that balance competing drives, such as the pursuit of essential rewards and the avoidance of potential threats (*1*, *2*). In real-world scenarios, food and other resources often co-occur with danger-predictive cues, forcing animals to suppress their innate or learned defensive reactions to obtain vital outcomes. These approach-avoidance decisions are among the most complex that an organism can face, requiring animals to integrate past aversive experiences, anticipate future threats, and value rewards (*3*–*6*). In humans, approach-avoidance decisions range from everyday risks to existential dilemmas and are commonly impaired under conditions such as post-traumatic stress disorder, generalized anxiety, and depression, where individuals struggle to evaluate options in emotionally uncertain contexts (*7*–*9*). Understanding the neural basis of these motivational conflicts is critical for elucidating how the brain guides adaptive behavior when a potential danger is anticipated. A key component of decision-making is behavioral restraint: The ability to withhold reward-seeking actions when doing so may incur a cost, such as a potential threat (*10*). This internal restraining of behavior based on remembered danger lies at the core of adaptive responses under motivational conflict; however, the neural mechanisms supporting this capacity remain unclear.

In the context of motivational conflict, whereas the amygdala encodes threat-related information and orchestrates defensive behaviors (*11*–*13*), striatal circuits, including the nucleus accumbens (NAc) and ventral pallidum (VP), integrate threat and reward signals to shape motivated actions (*14*–*17*). In parallel, the medial prefrontal cortex (mPFC) in rodents contributes to action selection under conflicting motivational demands, partly through regulatory control of subcortical circuits. In particular, interactions between the mPFC and NAc have been implicated in approach-avoidance decision-making (*18*–*23*). However, whether specific prefrontal-striatal projections mediate memory-guided restraint in response to learned threats remains unknown.

To address this gap, we used the crossing-mediated conflict (CMC) task, an ethologically relevant foraging paradigm in which rats decide whether to cross a shock-paired grid to retrieve food, guided by auditory and visual cues signaling a reward or danger (*17*, *24*). This paradigm models ethologically relevant decisions in which reward pursuit must be weighed against learned threat signals, requiring animals to regulate defensive responses while pursuing food. Using a multimodal approach, including pharmacological inactivation, exploratory single-unit recordings, c-Fos mapping, and optogenetic manipulation, we dissected the role of mPFC-NAc circuits in this process. We found that both the prelimbic cortex (PL) region of the mPFC and the anterior NAc core (aNAcC) of the striatum were selectively recruited and necessary for behavioral restraint during conflict. In general, optogenetic silencing of the PL→aNAcC projection diminished this restraint, even in the presence of threat-predictive cues, without impairing memory or basic motivation. These findings reveal a corticostriatal pathway through which PL control of the aNAcC contributes to adaptive restraint during reward seeking under learned threat conflict.

## RESULTS

**Fig. 1.**
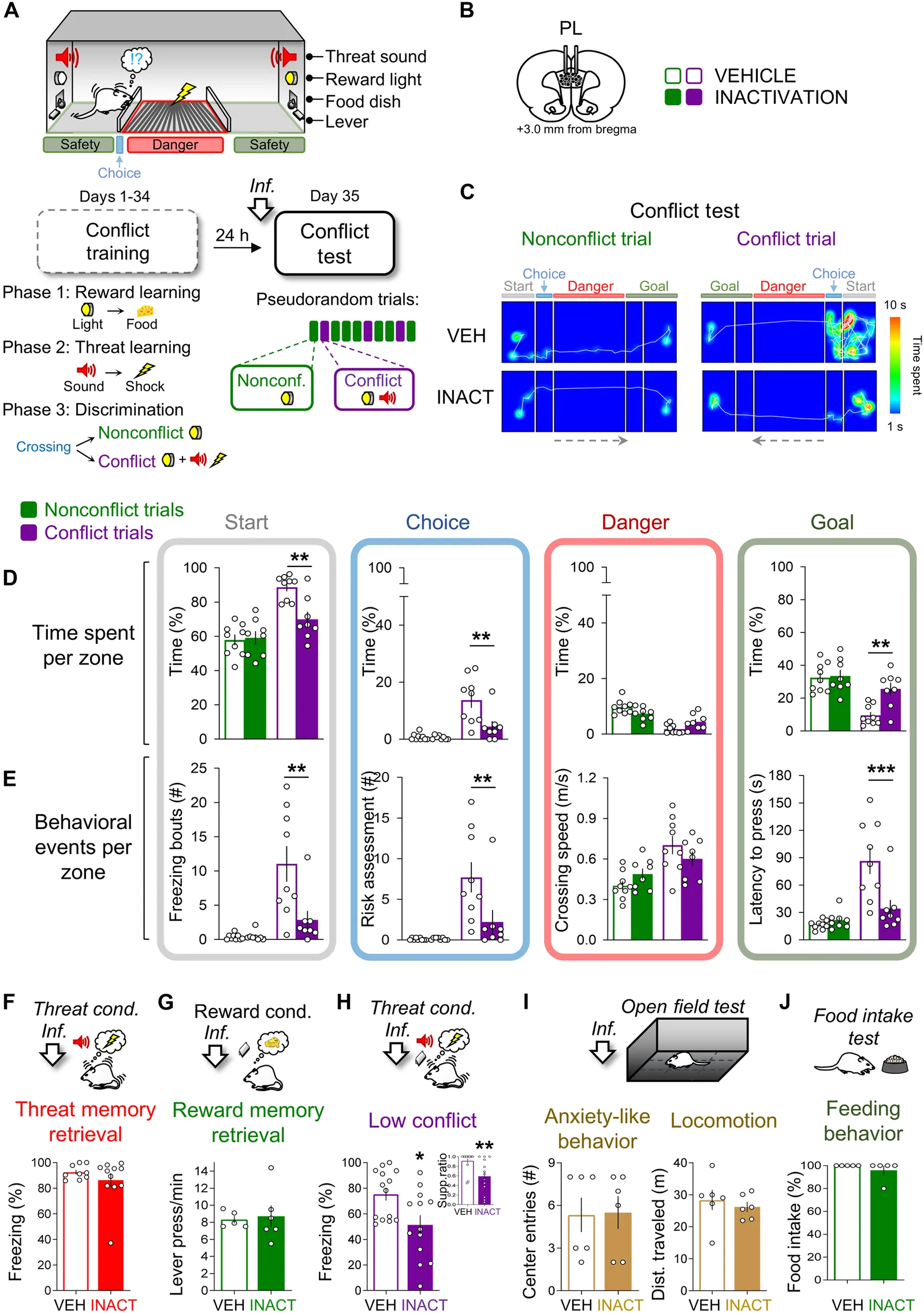
PL inactivation abolishes defensive responses and accelerates reward approach under learned threat. (**A**) Top: Schematic representation of the CMC task. Rats foraged between feeding zones under nonconflict trials (reward cue only) and conflict trials (reward cue plus threat cue). Bottom: Training comprised (i) reward learning via light cue in a safe zone, (ii) threat learning via white noise in a danger zone, and (iii) discrimination between conflict (light + noise) and nonconflict (light only) trials. In the conflict test, rats decided whether to cross an electrified grid to obtain food. h, hours. (**B**) Cannula placement in the PL. Before testing, rats received PL infusions of saline (VEH, *n* = 9) or muscimol+baclofen (INACT, *n* = 8). (**C**) Representative movement tracking and heatmaps from VEH and INACT rats. (**D**) PL inactivation decreased the time spent in the start and choice zones and increased goal zone time during conflict trials, without affecting nonconflict trials. (**E**) In conflict trials, PL inactivation reduced freezing and risk assessment in the start zone and decreased the latency to cross into the goal zone and press the lever. (**F** and **G**) No effect on freezing or lever pressing in the isolated threat (VEH, *n* = 9; INACT, *n* = 11) or reward (VEH, *n* = 5; INACT, *n* = 6) retrieval tests. (**H**) Under low conflict, PL inactivation reduced freezing and the suppression ratio (VEH, *n* = 14; INACT, *n* = 13). (**I** and **J**) No differences in anxiety-like behavior or feeding (open field test: VEH, *n* = 6; INACT, *n* = 6; food intake test: VEH, *n* = 5; INACT, *n* = 5). Data are presented as means ± SEM. *P < 0.05; **P < 0.01; ***P < 0.001, Bonferroni post hoc after a two-way mixed-design ANOVA or Student’s *t* test. Statistics are provided in the dataset deposited in Dryad (https://doi.org/10.5061/dryad.7wm37pw7s).

Rats were trained in the CMC task to discriminate between safe (nonconflict) and risky (conflict) crossings to obtain food rewards. They learned to associate distinct cues with opposing outcomes, such as food or foot shock, and used this information to guide decisions in either safe or threatening contexts (Fig. 1A). The training protocol comprised three phases: (i) reward learning, with a light cue signaling food availability, (ii) threat learning, with a white noise cue paired with a foot shock, and (iii) discrimination training between nonconflict (light only) and conflict (light plus noise) trials. In the final test, no shocks were delivered, ensuring that the decisions reflected memory-guided integration of the learned reward and threat associations. The representative behaviors during this phase are shown in movie S1.

### The PL, but not the IL, enables restraint under learned threat

The mPFC has been implicated in the regulation of defensive responses to threat-predictive cues, with the infralimbic cortex (IL) suppressing such responses and the PL facilitating them (*25*, *26*). On the basis of this framework, we initially hypothesized that the IL might be necessary to override defensive responses during reward-seeking under conflict. However, pharmacological inactivation of the IL before a memory-guided conflict test did not alter behavior; rats spent similar time across spatial zones (start, choice, danger, and goal) and showed unaffected measures of defensive behavior, including freezing, risk assessment, crossing speed, and lever press latency (fig. S1, A to D). These results suggest that the IL is not required to restrain behavior when threat-related memories must be weighed against current goals.

In contrast, PL inactivation markedly impaired performance during the conflict trials (Fig. 1, B to E). Rats spent significantly less time in the start zone and showed reduced freezing and risk assessment, along with shorter latencies to press the lever in the goal zone. Notably, the time spent and behavioral events in the danger zone were unaffected, indicating that PL inactivation did not cause generalized disinhibition or impair the ability to discriminate between zones. Instead, behavior during conflict trials became indistinguishable from that during nonconflict trials, suggesting that PL inactivation abolished behavioral restraint normally imposed by the learned threat. Trial-by-trial analyses showed that the PL inactivation effect was present from the first conflict trial (fig. S2) and was not explained by conflict-trial order [generalized estimating equation (GEE) model; start: β = −0.17, *P* = 0.15, 95% confidence interval (CI), [−2.77, 0.43]; choice: β = −0.30, *P* = 0.74, 95% CI, [−2.17, 1.55]; goal zones: β = 1.02, *P* = 0.14, 95% CI, [−0.34, 2.39]; freezing: β = 0.41, *P* = 0.38, 95% CI, [−0.52, 1.36]; risk assessments: β = 0.60, *P* = 0.17, 95% CI, [−0.27, 1.47]; latency to press: β = 3.03, *P* = 0.35, 95% CI, [−3.37, 9.44]]. To rule out nonspecific effects, we performed a series of control experiments using separate cohorts. PL inactivation did not alter freezing to a conditioned tone or instrumental food-seeking behavior (Fig. 1, F and G) nor did it affect anxiety-like behavior, spontaneous locomotion, or food consumption (Fig. 1, I and J). Furthermore, in a low-conflict version of the task, in which rats could press a lever for reward in the presence of a threat-predictive cue without crossing the shock-paired danger zone, PL inactivation reduced freezing and the suppression ratio (Fig. 1H), suggesting that the PL supports adaptive restraint even under low-demand conditions.

These results demonstrated that PL activity, but not IL activity, is selectively required to suppress reward-seeking behavior when threat-predicting cues are present in the environment. This effect cannot be explained by impairment in memory, motivation, or motor performance. Rather than directly mediating defensive responses, the PL appears to integrate competing motivational signals to guide behavior under conflict, consistent with prior reports implicating this region in cost-benefit evaluation, cognitive control, and flexible decision-making during approach-avoidance conflict (*27*–*34*). Thus, the PL supports context-sensitive restraint when memory-based threat signals must be overcome in the pursuit of rewards, setting the stage for downstream circuits that may implement cortical decisions.

**Fig. 2.**
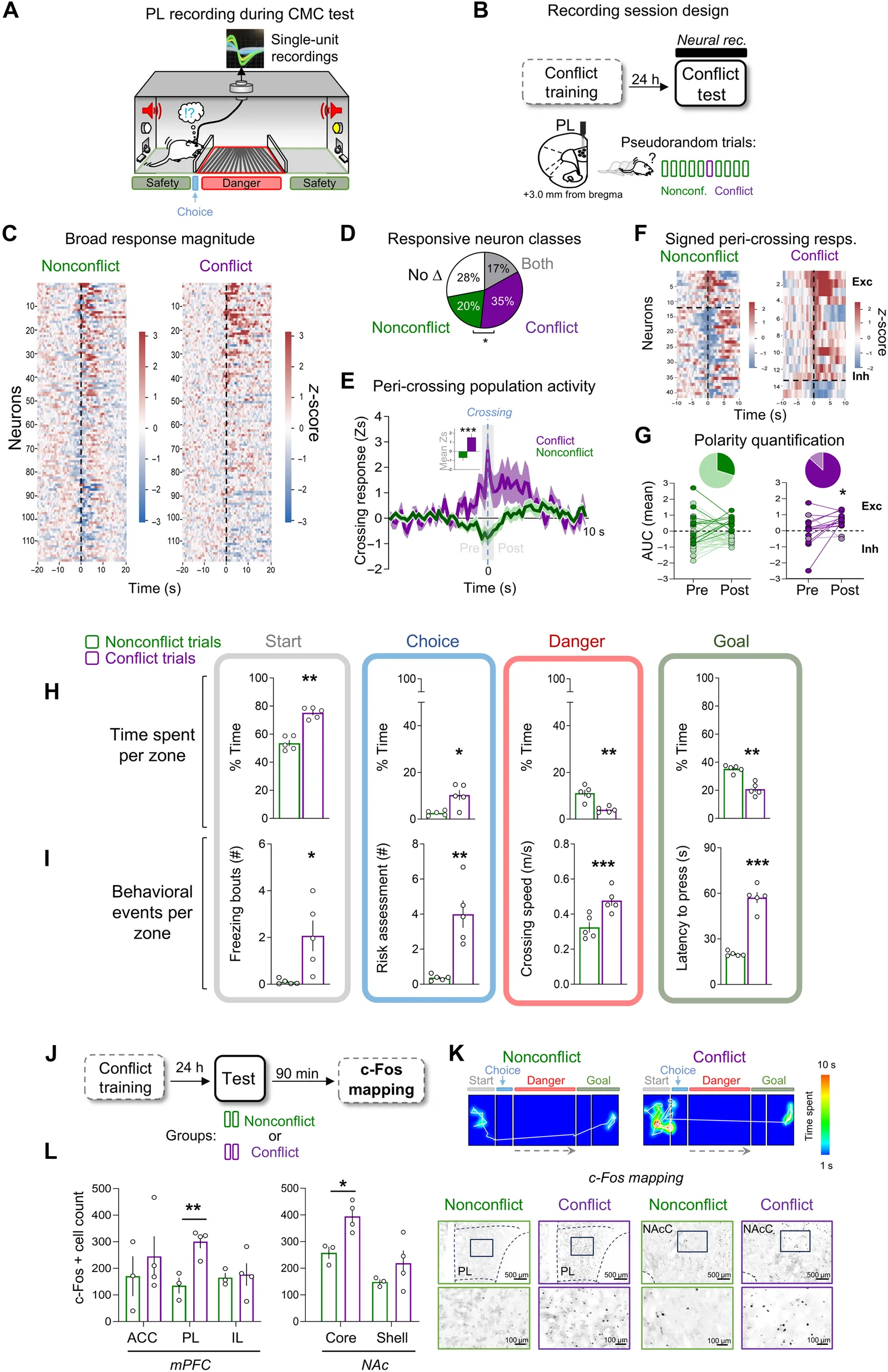
PL neuronal dynamics and c-Fos mapping identify selective recruitment of a PL-NAcC circuit under threat. (**A**) Schematic of single-unit recordings in the PL during the conflict test (*n* = 5 rats). (**B**) Electrode placement and trial structure. (**C**) Heatmaps of *z*-scored broad-response neuronal activity aligned to crossing onset during nonconflict and conflict trials. (**D**) Proportions of PL neurons (*n* = 118) classified as conflict-responsive (*n* = 42), nonconflict-responsive (*n* = 23), both (*n* = 20), or unmodulated (*n* = 33). (**E**) Average normalized firing rates of conflict (*n* = 15) and nonconflict (*n* = 42) neurons. Inset: Mean activity (±600 ms) of conflict and nonconflict neurons. Zs, *z*-scores. (**F**) Heatmaps of excitatory and inhibitory pericrossing neuronal responses. (**G**) The AUC confirmed excitation in conflict-excited neurons versus baseline but no modulation in nonconflict neurons. Pie charts show the excitation/inhibition in conflict (87%/13%) and nonconflict (29%/71%). (**H** and **I**) Behavioral profile of recorded rats during nonconflict and conflict trials. (**J**) Rats (nonconflict, *n* = 3; conflict, *n* = 4) were perfused 90 min after the test for c-Fos mapping. (**K**) Representative trajectories and heatmaps during conflict and nonconflict trials. (**L**) c-Fos mapping revealed higher PL and NAcC activity in conflict rats; no differences in ACC, IL, or NAcS. Representative PL and NAcC images shown. Scale bars, 500 μm and 100 μm (inset). Data are means ± SEM. *P < 0.05; **P < 0.01; ***P < 0.001, Student’s *t* test or Wilcoxon signed-rank test. Heatmaps use a diverging *z*-score color scale centered at zero to distinguish negative and positive pericrossing activity. Statistics are provided in the dataset deposited in Dryad (https://doi.org/10.5061/dryad.7wm37pw7s).

Given that PL activity is required for behavioral restraint under conflict conditions, we next examined whether PL neurons were preferentially engaged under such conditions. We recorded extracellular single-unit activity in well-trained rats performing the conflict test (Fig. 2, A and B), focusing on responses time-locked to the moment animals crossed from the choice zone to the danger zone. Heatmaps of all recorded neurons showed the full response distribution (Fig. 2C), whereas signed-responder heatmaps illustrated the direction of pericrossing modulation among neurons classified as excitatory or inhibitory within the decision-period window (Fig. 2F). We first classified all recorded neurons according to broad task responsiveness across the −20- to +20-s pericrossing window. This analysis identified neurons responsive during conflict trials, nonconflict trials, both trial types, or neither (Fig. 2D). We then performed a separate signed pericrossing analysis to determine whether neurons showed excitatory or inhibitory modulation around crossing onset within the decision-period window. The signed analysis is shown with signed-responder heatmaps and the corresponding ±600-ms quantification in Fig. 2 (E and F). At the population level, conflict-responsive neurons displayed pronounced transient excitation centered on the crossing, typically preceded by ramping activity, whereas nonconflict-responsive neurons showed slower and less sharply tuned patterns (Fig. 2E). Quantification of the mean *z*-score activity (±600 ms) confirmed significantly higher firing in conflict neurons (Fig. 2E, inset). Area under the curve (AUC) analyses showed that most conflict-responsive neurons increased firing after the decision point, whereas nonconflict neurons were more often inhibited. This divergence was evident in both the proportion of excitatory and inhibitory responses (pie charts) and the direction of individual pre/post-trajectories (color-coded lines) (Fig. 2G). Similar neural dynamics were observed within the shorter peri-event window (±300 ms) but not within the longer ±900-ms window (fig. S3, A to D), suggesting that PL neuronal activity was tightly time locked to the crossing event. These neuronal dynamics were accompanied by distinct behavioral patterns: During conflict trials, rats spent more time in the start and choice zones and less time in the danger and goal zones (Fig. 2H) and showed increased freezing, greater risk assessment, and longer lever press latencies, despite rapid danger zone crossings (Fig. 2I). Locomotor speed in the start (safe) zone did not differ between conditions (fig. S3E), arguing against generalized locomotor differences as an explanation for the decision-aligned PL activity.

Given the known role of the NAc in mediating reward and aversion (*35*, *36*), we assessed whether other brain regions were recruited during this form of behavioral restraint by quantifying c-Fos expression in rats exposed to either conflict or nonconflict trials (Fig. 2J). The behavioral profile of the c-Fos cohort confirmed that conflict animals showed increased start/choice zone occupancy, increased defensive responding, and longer reward-approach latencies relative to nonconflict animals, with no corresponding group difference in crossing speed (fig. S3, G and H). c-Fos levels in the PL increased relative to controls, with no differences in the IL or anterior cingulate cortex (ACC). In the NAc, conflict exposure selectively increased c-Fos expression within the core (NAcC) but not in the shell (NAcS) (Fig. 2L). These c-Fos data provide supportive evidence that conflict engages the PL and NAcC, but they do not by themselves distinguish conflict resolution from increased defensive responding. We therefore interpret this mapping as hypothesis-generating and complementary to the causal manipulations.

### The aNAcC mediates restraint under learned threat

Our c-Fos mapping revealed selective activation of the NAcC, but not the NAcS, during behavioral restraint under conflict, leading us to functionally target the NAcC. Pharmacological inactivation of the entire NAcC before the conflict test produced divergent behavioral outcomes (fig. S4A). Some rats showed reduced latencies to press under conflict, whereas others displayed generalized suppression of approach behavior across trials (fig. S4B). On the basis of previous reports of functional heterogeneity along the anteroposterior (AP) axis of the accumbens (*37*), we applied an unsupervised clustering approach, using cannula placement in the NAcC. NAcC cannula placements were classified using hierarchical agglomerative clustering applied to +1.5, +1.8, +2.0, +2.3, +2.5, and +2.8 mm. Coordinates were defined as the center of the cannula and injector relative to the bregma. The analysis used Euclidean distance and Ward linkage criteria, with the number of clusters set to *k* = 2 to test the anatomically motivated AP subdivision of the NAcC. Clusters were then labeled anterior or posterior based on their mean AP coordinate, and behavioral measures were subsequently plotted against the AP coordinate as a complementary continuous analysis. To directly test whether the behavioral heterogeneity followed the AP axis, we plotted key behavioral effects as a function of the cannula AP coordinate (fig. S4, D to F). More posterior NAcC (pNAcC) placements were associated with longer reward-approach latencies in both conflict and nonconflict trials, whereas more anterior placements were associated with reduced restraint-related behavior during conflict. The same AP coordinate analysis in the PL (fig. S4, G to K) did not reveal a comparable anatomical-behavioral relationship, supporting the interpretation that the NAcC effect reflects rostrocaudal functional heterogeneity rather than generic placement variability. Overall, these findings prompted us to analyze the aNAcC and pNAcC separately in the following experiments, beginning with the aNAcC, which is the region associated with reduced restraint in cluster analysis.

**Fig. 3.**
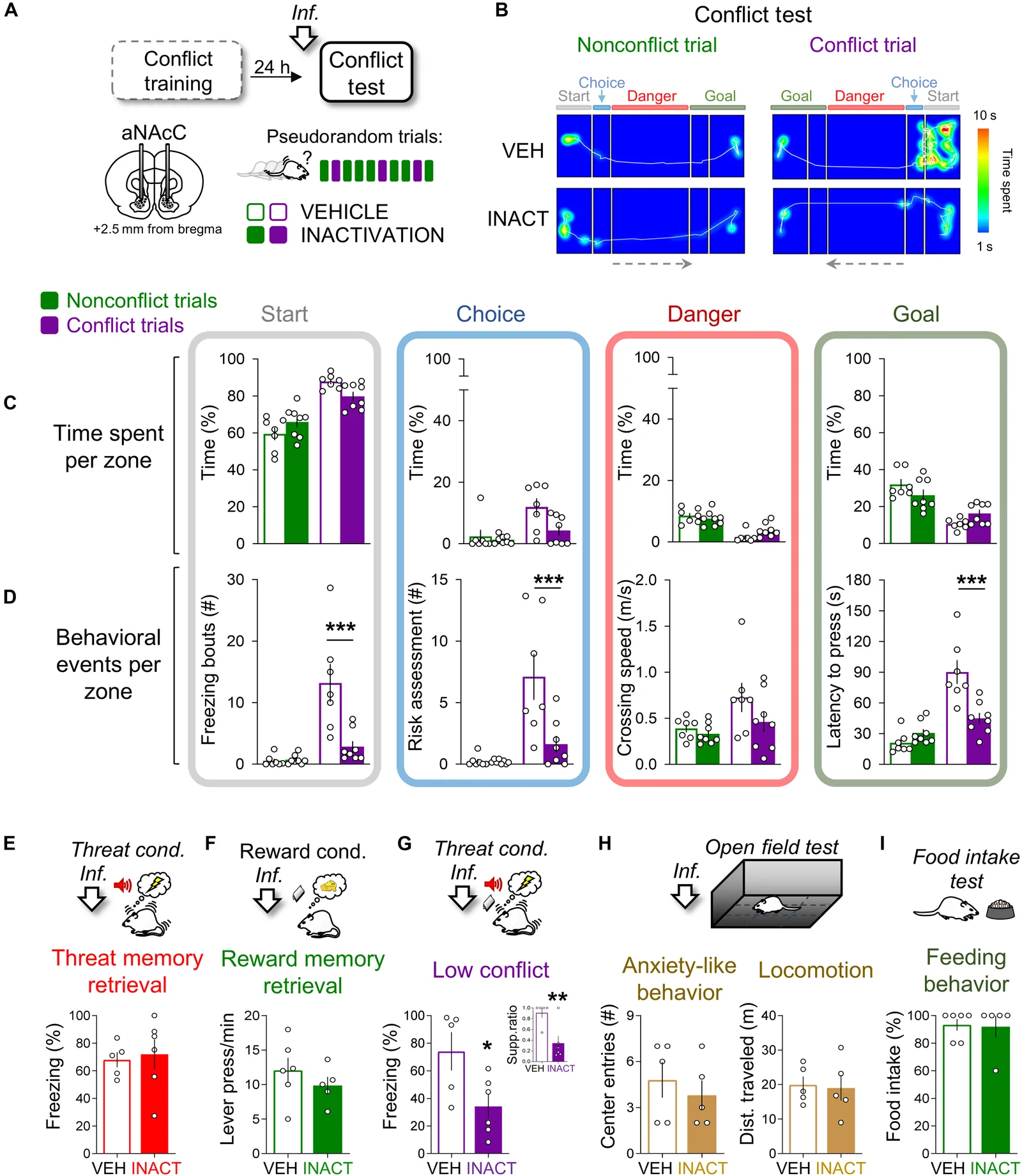
aNAcC inactivation reduces defensive responses without affecting threat or reward memory. (**A**) Cannula placements in the aNAcC. Rats received vehicle (VEH, *n* = 7) or muscimol+baclofen (INACT, *n* = 8) before the conflict test. (**B**) Representative movement tracking and heatmaps from VEH and INACT rats during the first two consecutive nonconflict and conflict trials. (**C**) aNAcC inactivation did not significantly affect the time spent in the start, choice, danger, or goal zones. (**D**) In conflict trials, inactivation reduced freezing and risk assessment in the start zone and decreased the latency to cross into the goal zone and press the lever. (**E** and **F**) No effect on freezing or lever pressing in the isolated threat (VEH, *n* = 5; INACT, *n* = 6) or reward (VEH, *n* = 6; INACT, *n* = 5) retrieval tests. (**G**) Under low conflict, inactivation decreased freezing and the suppression ratio (VEH, *n* = 5; INACT, *n* = 6). (**H** and **I**) No differences in anxiety-like behavior or locomotion in the open field (VEH, *n* = 5; INACT, *n* = 5) or feeding in the free food intake test (VEH, *n* = 6; INACT, *n* = 5). Data are means ± SEM. *P < 0.05; **P < 0.01; ***P < 0.001, Bonferroni post hoc after a two-way mixed-design ANOVA or Student’s *t* test. Statistics are provided in the dataset deposited in Dryad (https://doi.org/10.5061/dryad.7wm37pw7s).

To determine the specific contribution of the aNAcC, we pharmacologically inactivated this region before the conflict test. aNAcC inactivation did not affect the time spent in any of the spatial zones (Fig. 3, A to C). However, during conflict trials, it significantly decreased freezing and risk assessment, resulting in shorter lever press latencies (Fig. 3D). These changes resembled the effects of PL inactivation, although they were somewhat milder and occurred without impairing spatial navigation or trial discrimination, as indicated by the preserved crossing speeds. Trial-by-trial analyses showed that aNAcC inactivation reduced restraint-related behavior from the first conflict trial, arguing against within-session extinction as the primary explanation (fig. S5). The order of conflict-trial presentation did not influence the effects observed in freezing (β = 0.92, *P* = 0.25, 95% CI, [−0.67, 2.53]) and latency to press (β = 4.17, *P* = 0.15, 95% CI, [−1.52, 9.87]) but did so during risk assessment (β = 1.34, *P* = 0.01, 95% CI, [0.26, 2.42]). These results suggest that aNAcC activity helps suppress premature reward pursuit when a threat memory is present, even during the initial exposure to conflict. To further assess specificity, we tested aNAcC-inactivated rats using control tasks. Freezing to a conditioned tone and instrumental food-seeking behavior were unaffected (Fig. 3, E and F), indicating that basic threat and reward memories remained intact. However, in a low-conflict retrieval task in which food-seeking occurs without crossing, but in the presence of a threat-predictive cue, aNAcC inactivation reduced freezing and the suppression ratio (Fig. 3G), supporting its role in modulating behavior, even when motor demands are minimal. Anxiety-like behavior, locomotion, and feeding also remained unchanged (Fig. 3, H and I). Together, these findings identify the aNAcC as a critical node for implementing behavioral restraint during motivational conflict. Its contribution parallels that of the PL, supporting the idea that prefrontal and striatal regions interact to regulate adaptive decision-making when threat and reward information must be integrated.

### The pNAcC promotes reward-seeking regardless of threat

**Fig. 4.**
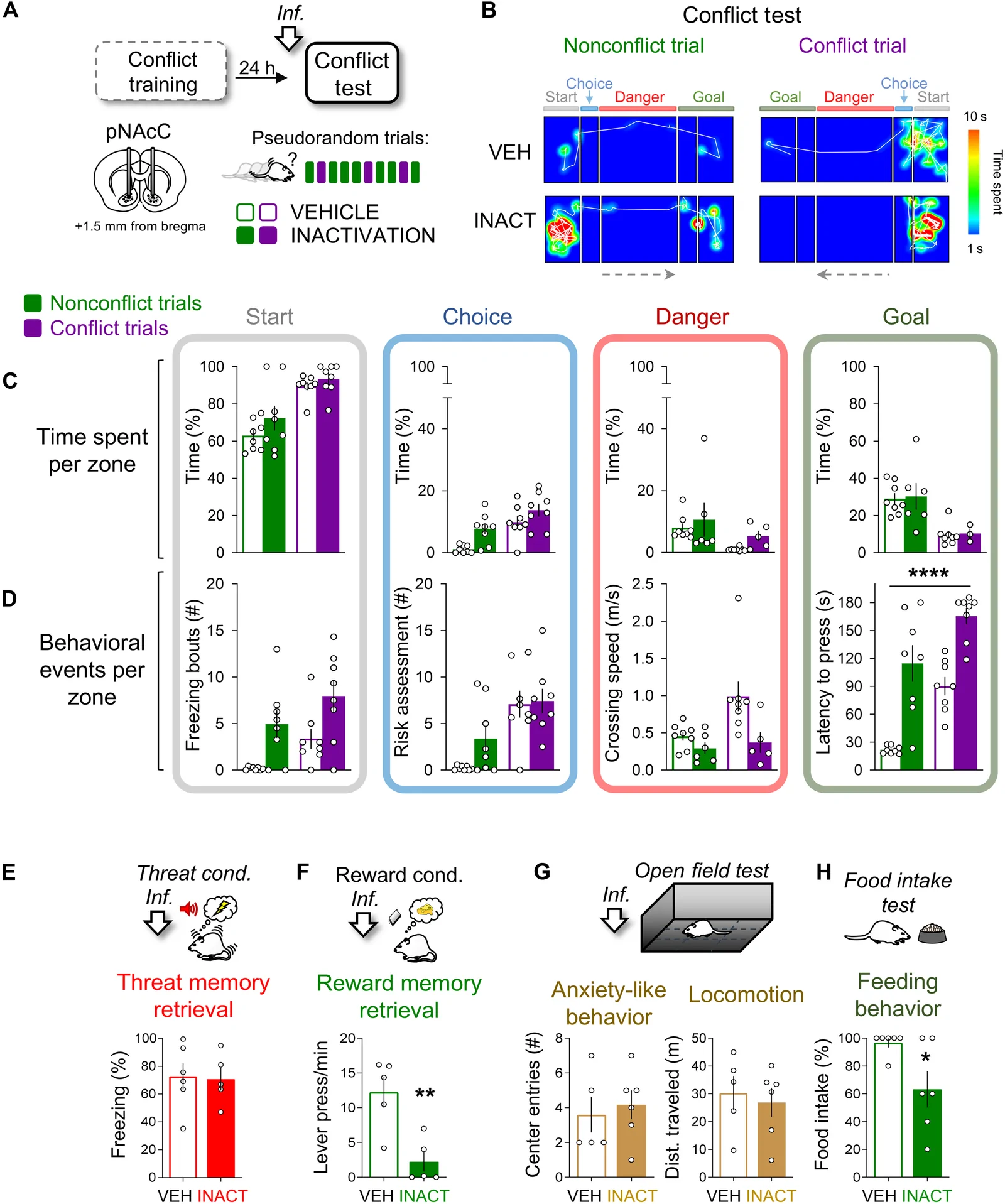
pNAcC inactivation impairs general reward pursuit regardless of threat signals. (**A**) Cannula placements in the pNAcC. Rats received vehicle (VEH, *n* = 8) or muscimol+baclofen (INACT, *n* = 8) before the conflict test. (**B**) Representative movement tracking and heatmaps from VEH and INACT rats during nonconflict (reward cue only) and conflict (reward cue + threat cue) trials. (**C**) pNAcC inactivation did not significantly alter the time spent in start, choice, or goal zones. (**D**) pNAcC inactivation showed increased latency to cross into the goal zone and press the lever, revealing a significant main effect of group. (**E**) No effect on freezing in isolated threat memory retrieval (VEH, *n* = 6; INACT, *n* = 5). (**F**) In reward memory retrieval, inactivation reduced lever pressing (VEH, *n* = 5; INACT, *n* = 5). (**G**) No differences in anxiety-like behavior or locomotion were observed in the open field (VEH, *n* = 5; INACT, *n* = 6). (**H**) Inactivation reduced feeding in the free food intake test (VEH, *n* = 6; INACT, *n* = 6). Data are means ± SEM. *P < 0.05; **P < 0.01, ****P < 0.0001, Bonferroni post hoc after a two-way mixed-design ANOVA or Student’s *t* test. Statistics are provided in the dataset deposited in Dryad (https://doi.org/10.5061/dryad.7wm37pw7s).

Next, we examined the role of the pNAcC, identified by cluster analysis as associated with enhanced behavioral restraint. Pharmacological inactivation of this region before the conflict test (Fig. 4A) did not alter time allocation across spatial zones, defensive responses (freezing and risk assessment), or crossing speed during conflict or nonconflict trials (Fig. 4, B to D). However, a main effect of group revealed significantly increased latency to press the lever for food, regardless of trial type. Notably, 40% of the pNAcC-inactivated rats failed to cross the arena within 180 s, indicating a reduction in baseline incentive motivation rather than a trial type–specific effect. This motivational deficit persisted even under simplified conditions, in which rats were placed directly in the goal zone without the need to cross. Trial-by-trial analysis showed that pNAcC inactivation increased time spent in the goal zone and increased freezing bouts and latency to press in most nonconflict trials but not conflict trials, suggesting that pNAcC inactivation preferentially affected reward-seeking behavior in the absence of threat (fig. S6). In this context, pNAcC-inactivated animals were slower to press the lever (VEH: 4 s, INACT: 54 s; *t*(14) = 2.76, *P* = 0.01), similar to the reduced reward pursuit observed with VP inactivation (*17*). This suggests that the pNAcC and VP may work in concert to sustain an appetitive drive independent of the threat context.

To further confirm that the observed behavioral changes reflected motivational rather than mnemonic deficits, we conducted additional tests. For instance, pNAcC inactivation did not impair freezing responses to the conditioned tone (Fig. 4E), indicating an intact threat memory. However, reward-related behaviors were consistently reduced: pNAcC-inactivated rats pressed less for food (Fig. 4F) and consumed less food in the home cage (Fig. 4H), similar to what was observed in the caudal NAcS in a previous report (*37*). The measures of anxiety-like behavior and locomotion remained unchanged (Fig. 4G). Together, these results indicate that the pNAcC is critical for sustaining goal-directed reward seeking, independent of threat or behavioral restraint, underscoring the functional dissociation along the rostrocaudal axis of the NAcC.

### The PL→aNAcC pathway enables adaptive restraint under learned threat

**Fig. 5.**
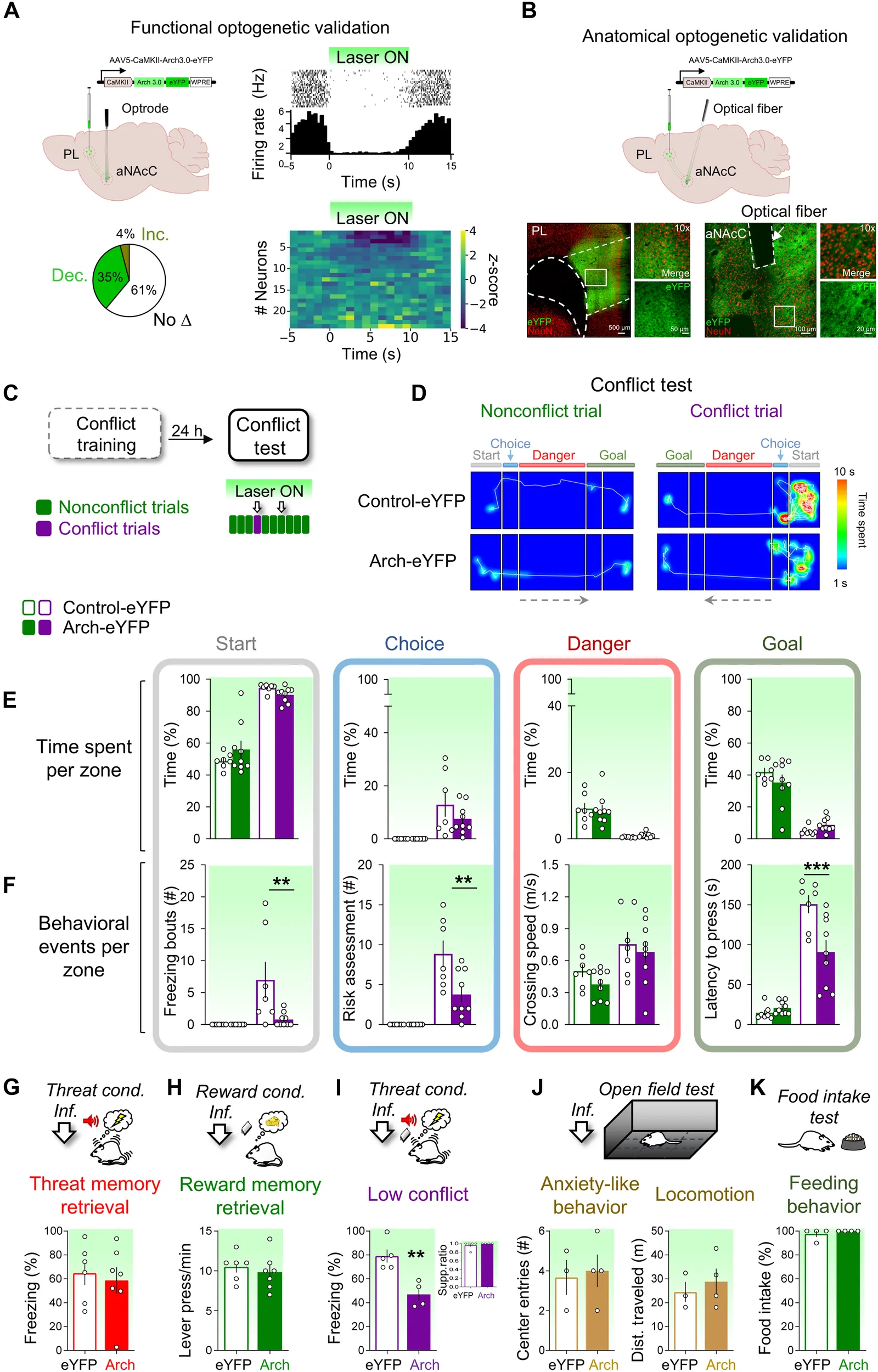
Silencing the PL→aNAcC projection reduces defensive responses and facilitates reward approach. (**A**) Top left: Schematic showing the viral injection of AAV5-CaMKII-Arch-EYFP in the PL and optrode implantation in the aNAcC (*n* = 5 rats). Top right: Raster plot of a representative unit during photoinhibition (laser on). Bottom left: Percentage of PL→aNAcC neurons (*n* = 23) modulated during photoinhibition. Bottom right: Color map (*z*-score) of neural responses aligned to laser onset. (**B**) Top: Schematic representation of the viral infection in the PL and bilateral optical fiber in the aNAcC. Bottom: Micrographs showing the viral expression in the PL and axonal projections to the aNAcC (arrow: fiber track). (**C**) During the conflict test (Control-eYFP, *n* = 7; Arch-eYFP, *n* = 9), rats underwent two crossing trial blocks: laser ON followed by laser OFF (figs. S7 and S8). (**D**) Overlaid movement tracking and heatmaps from representative Control-eYFP and Arch-eYFP rats during nonconflict and conflict trials. (**E**) Photoinhibition of PL→aNAcC projections did not alter zone times during the conflict test. (**F**) Photoinhibition reduced freezing and risk assessment in the start zone and decreased the latency to reach the goal zone and press the lever during conflict trials. (**G** and **H**) Photoinhibition during the threat or reward tests (Control-eYFP, *n* = 6; Arch-eYFP, *n* = 7). (**I**) In the low-conflict test, photoinhibition decreased freezing without altering the suppression ratio (Control-eYFP, *n* = 5; Arch-eYFP, *n* = 4). (**J** and **K**) Photoinhibition did not affect anxiety-like behavior, locomotion (Control-eYFP, *n* = 3; Arch-eYFP, *n* = 4), or food intake (Control-eYFP, *n* = 4; Arch-eYFP, *n* = 4). Scale bars, 500 μm (PL) and 100 μm (aNAcC); insets: 50 μm (PL) and 20 μm (aNAcC). Data are means ± SEM. **P < 0.01; ***P < 0.001, Bonferroni post hoc after a two-way mixed-design ANOVA, or Student’s *t* test. Statistics are provided in the dataset deposited in Dryad (https://doi.org/10.5061/dryad.7wm37pw7s).

Our pharmacological inactivation findings demonstrated that both the PL and the aNAcC are necessary for suppressing food-seeking behavior in the presence of learned threat cues, suggesting that a prefrontal-striatal circuit supports behavioral restraint. To directly test the role of the PL→aNAcC projection, we used optogenetic tools to selectively inhibit or activate this pathway during the conflict task. We bilaterally infused the PL with an adeno-associated virus (AAV) encoding both archaerhodopsin (Arch) and enhanced yellow fluorescent protein (eYFP) under the control of the calcium- and calmodulin-dependent protein kinase II (CaMKII) promoter to target glutamatergic neurons. To validate the photoinhibition efficacy, we implanted a unilateral optrode into the aNAcC of rats. Photoinhibition of PL terminals in the aNAcC significantly reduced the firing rate of over one-third of the recorded neurons, confirming the effective suppression of PL input to the aNAcC (Fig. 5A). Anatomical verification confirmed robust eYFP expression in PL pyramidal neurons and dense terminal labeling in the aNAcC, consistent with targeted pathway expression (Fig. 5B).

For behavioral testing, we expressed either Arch or eYFP in PL neuronal bodies and bilaterally implanted optical fibers into the aNAcC of rats. Rats were tested in two consecutive trial blocks: one conflict and one nonconflict trial with photoinhibition (laser-on; Fig. 5C), followed by the same trial types without stimulation (laser-off; fig. S7A). PL→aNAcC photoinhibition selectively reduced defensive responses (freezing in the start zone and risk assessment in the choice zone) during conflict trials, with no effect on nonconflict behavior. These changes were accompanied by shorter latencies to reach the goal zone and press the lever. Trial discrimination and navigation remained intact, as indicated by the preserved crossing speed and spatial zone occupancy (Fig. 5, E and F). In laser-off trials, Arch-expressing rats exhibited longer latencies than eYFP controls, possibly reflecting the delayed extinction of threat-associated memory (fig. S7, B and C). Because the photoinhibition experiment used a fixed laser-on/laser-off sequence, we analyzed stimulated conflict trials, matched unstimulated trials (figs. S7 and S8), and trial-order effects separately (GEE model; freezing: β = −0.39, *P* = 0.54, 95% CI, [−1.68, 0.89]; risk assessments: β = 0.55, *P* = 0.44, 95% CI, [−0.86, 1.98]; latency to press: β = 4.59, *P* = 0.46, 95% CI, [−7.79, 16.98]). To rule out general deficits in memory or motivation, threat and reward retrieval were assessed using separate tasks. PL→aNAcC photoinhibition did not alter freezing to a conditioned tone or lever pressing for food (Fig. 5, G and H), indicating intact cue-reward and cue-threat associations. In a low-conflict retrieval test, photoinhibition reduced freezing during laser-on tone presentation but not during the subsequent laser-off tone (fig. S7F), confirming a transient and specific effect. However, the suppression ratio remained unaltered during laser-on tone presentation, suggesting that photoinhibition was insufficient to elicit reward-seeking behavior, despite the suppression of passive defensive reactions in the low-conflict test (Fig. 5I). Thus, defensive immobility and instrumental suppression should be interpreted as related but dissociable components of adaptive restraint rather than interchangeable measures of a single behavioral process. Anxiety-like behavior, locomotion, and feeding were unaffected (Fig. 5, J and K).

To assess whether PL→aNAcC activity is sufficient to influence restraint, we expressed channelrhodopsin-2 (ChR2) or eYFP in PL neuronal soma and optically stimulated aNAcC terminals during the conflict task (fig. S9A). Contrary to the induction of selective restraint, photoactivation disrupted behavior more broadly; it increased crossing latency in both conflict and nonconflict trials without altering defensive behaviors (fig. S9, C to F). Despite using the stimulation parameters used in a previous report (*23*), the trajectory data revealed disorganized time allocation across zones (fig. S9B), suggesting that activation of the pathway impairs goal-directed behavior, regardless of the threat context. Together, these findings indicate that the PL→aNAcC projection is necessary for expressing adaptive behavioral restraint under threat, but that overactivation disrupts goal pursuit, highlighting the need for balanced activity in this corticostriatal pathway for decision-making under motivational conflict.

### The aNAcC contributes to restraint under innate threat independently of the PL

Our previous results demonstrated that PL→aNAcC projection is necessary to restrain reward-seeking behavior in the presence of learned threat cues. However, in natural environments, animals must also be regulated in the presence of innate threats, such as bright and exposed spaces that signal danger during foraging. To examine whether the same prefrontal-striatal circuits support restraint under innate threat, we tested the effects of PL-aNAcC inactivation as well as PL→aNAcC photoinhibition using the foraging-mediated innate conflict task (*24*).

**Fig. 6.**
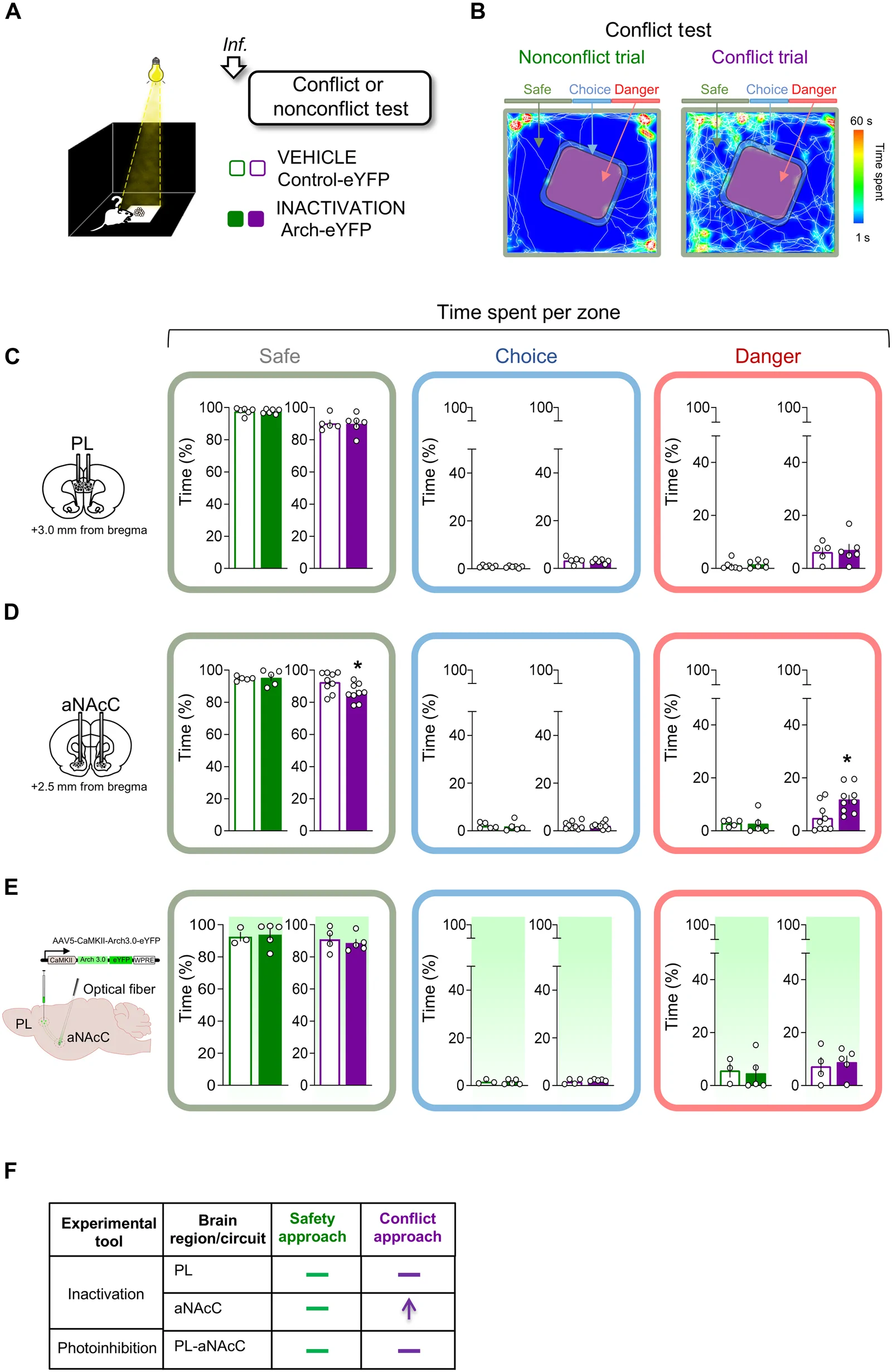
aNAcC, but not PL or PL→aNAcC projections, supports restraint under innate threat. (**A**) Rats were tested in a dark arena where they foraged for food in a brightly lit center (conflict test) versus an unlit center (nonconflict test). Before testing, they received vehicle (VEH) or muscimol+baclofen (INACT) infusions into the PL or aNAcC. (**B**) Overlaid movement tracking and heatmaps from a representative saline-infused rat in nonconflict and conflict trials. (**C**) Injection sites in the PL. Inactivation did not affect time spent in start or danger zones (VEH, *n* = 6; INACT, *n* = 6 in nonconflict; VEH, *n* = 5; INACT, *n* = 6 in conflict). (**D**) Injection sites in the aNAcC. Inactivation reduced time in the start zone and increased time in the danger zone during conflict (VEH, *n* = 9; INACT, *n* = 9), with no effects in the nonconflict test (VEH, *n* = 5; INACT, *n* = 5). (**E**) Viral injection in the PL and bilateral fiber implantation in the aNAcC. Photoinhibition of PL→aNAcC projections did not alter zone occupancy in nonconflict (Control-eYFP, *n* = 3; Arch-eYFP, *n* = 5) or conflict (Control-eYFP, *n* = 4; Arch-eYFP, *n* = 5) tests. (**F**) Summary table of experimental tools (inactivation and photoinhibition) and the brain regions tested for each condition: safety approach (green) versus conflict approach (purple). Data are presented as means ± SEM. *P < 0.05, Student’s *t* test. Statistics are provided in the dataset deposited in Dryad (https://doi.org/10.5061/dryad.7wm37pw7s).

This task presents food at the center of a brightly lit open field, confronting the foraging drive with a natural aversive stimulus (Fig. 6A). The arena was divided into three zones: safe (periphery), dangerous (center), and choice (transition zone between them). Animals were tested in either conflict trials (food present) or nonconflict trials (no food present), with different groups used for each condition. Pharmacological inactivation of the PL or aNAcC was performed before testing. Representative movement traces and heatmaps illustrate the zone occupancy during both trial types (Fig. 6B). PL inactivation did not alter the time spent in any zone during conflict or nonconflict trials, suggesting that PL activity was not required to restrain foraging in response to innate threats (Fig. 6C). This result aligns with previous findings that PL inactivation does not modulate innate defensive responses such as those evoked by predator exposure (*38*). In contrast, aNAcC inactivation significantly reduced the time spent by rats in the safe zone and increased the time spent in the danger zone during conflict trials, with no effect on nonconflict trials (Fig. 6D). These results indicate that the aNAcC is necessary to constrain reward-seeking behavior when innate threat cues are present. The behavioral effects closely resemble those observed following systemic diazepam administration in the same task (*24*), suggesting that the aNAcC integrates conflicting motivational signals across different threat domains in the same way.

**Fig. 7.**
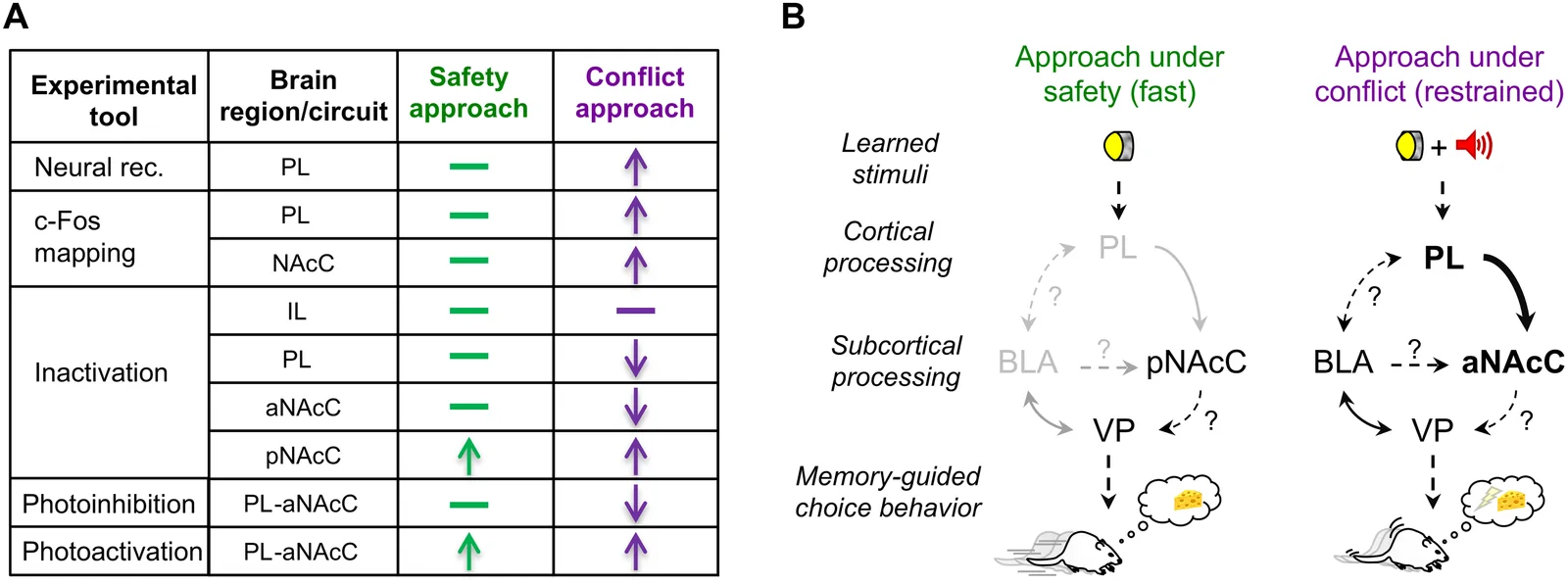
Experimental tools and proposed corticostriatal circuits for reward approach under safety versus conflict. (**A**) Summary table of experimental tools (neural recordings, c-Fos mapping, inactivation, photoinhibition, and photoactivation) and the brain regions tested for each condition: safety approach (green) versus conflict approach (purple). (**B**) Proposed circuit model. Left: Under safe conditions, reward-predictive cues elicit fast, unrestrained approach behavior, primarily driven by motivational signals through the pNAcC (this study) and VP (*17*). Right: When reward and threat cues co-occur, animals display a restrained approach marked by hesitation and risk assessment. This adaptive behavior depends on the PL and its projection to the aNAcC (this study), likely with modulatory inputs from the basolateral amygdala (BLA) to the VP (*17*). Together, these findings support a top-down corticostriatal contribution to learned threat–guided restraint during reward seeking while leaving the precise synaptic and cell type mechanisms for future work.

Given that previous studies have implicated prefrontal-striatal circuits in innate-avoidance behaviors (*39*, *40*), we evaluated whether the PL→aNAcC projection is also involved in innate threat restraint by expressing Arch or eYFP in the PL and bilaterally implanting optical fibers in the aNAcC (Fig. 6E). During the foraging conflict task, photoinhibition of the PL→aNAcC projection had no effect on zone occupancy under either conflict or nonconflict conditions. This suggests that, although the aNAcC contributes to innate threat modulation, its regulation may involve inputs from brain regions other than the PL. In summary, our findings indicate that the PL→aNAcC pathway is selectively engaged in learned threat contexts to mediate behavioral restraint, whereas innate threat regulation relies on distinct afferents to the aNAcC. These results highlight the flexible recruitment of neural circuits for adaptive behavioral control depending on the nature of the threat to the individual. The innate-threat results are summarized in Fig. 6F, indicating that the aNAcC contributes to restraint while its upstream pathways remain to be defined. The learned threat CMC circuit summary is presented separately in Fig. 7.

## DISCUSSION

Our results identify a distributed yet organized corticostriatal architecture that supports adaptive restraint under threat. Here, we use adaptive restraint operationally to refer to the threat-guided withholding or delay of reward approach, reflected across increased reward-approach latency, freezing, risk assessment, and reduced immediate goal-directed approach during learned threat conflict. The PL and aNAcC converge to withhold reward-seeking in the presence of learned threat cues, integrating competing motivational drives. In contrast, the pNAcC regulates general incentive motivation regardless of threat. Notably, the aNAcC also mediates restraint under innate threats, independent of PL input, indicating a shift in circuit recruitment depending on the threat source. These findings define a modular and flexible corticostriatal architecture for regulating decision-making under threat, with circuit engagement shaped by both threat type and task demands (Fig. 7). Together, these pathways contribute to top-down corticostriatal regulation of adaptive reward pursuit under threat conditions.

By combining pharmacological, electrophysiological, immunohistochemical, and optogenetic approaches in a seminaturalistic foraging task, we found that the PL and aNAcC are coengaged when rats decide whether to cross a danger-signaled zone for food. This ethologically grounded design, which allowed us to model decisions integrating memory, motivation, and internal conflict, captured components difficult to study in simpler organisms or constrained paradigms. Although prior studies have implicated the prefrontal cortex and NAc in reward processing (*41*–*45*) and aversive learning (*30*, *46*–*50*), our results uniquely demonstrate the dynamic interplay between the PL and aNAcC, specifically during conflict involving a learned threat. Heightened activity in these regions during conflict was required to preserve the hesitation or restraint typically seen in response to remembered danger, without impairing motor output, motivation, or memory. This aligns with evidence that prefrontal networks can represent emotionally salient associations to guide flexible behavior (*34*), and our findings identify a specific prefrontal-striatal pathway by which prior aversive learning exerts internal control over goal pursuit even in the absence of immediate danger, paralleling human findings on prefrontal-amygdala regulation of fear and reward (*51*, *52*).

Within this pathway, the PL emerges as a key hub in adaptive restraint. Under conflict conditions, both single-unit and c-Fos measurements demonstrated enhanced PL activity relative to nonconflict trials. Earlier research suggested a generalized role of the PL in fear expression (*26*, *27*, *38*, *42*, *47*, *53*–*55*); our data point to a more specific function: Modulating approach behavior when learned threats must be overcome to obtain a reward. PL inactivation selectively disrupted hesitation during conflict without affecting nonconflict performance or simpler Pavlovian and instrumental behaviors. Recordings revealed temporally precise, conflict-specific activity—often ramping before zone entry, peaking sharply at the decision point, and returning rapidly to baseline—that was overrepresented relative to chance. Together with comparable start zone speed across conditions, these temporally locked dynamics are most consistent with a context-dependent action-selection signal rather than generalized locomotor drive. These patterns, together with c-Fos data, argue against a role in general arousal or motor control, instead linking PL activity to a discrete signal for behavioral restraint. PL inactivation abolished defensive responses and sped up approach, complementing prior reports on approach-avoidance regulation (*30*, *32*) and supporting a context-dependent role engaged during cue-driven but not innate threat (*56*). This role should not be interpreted as specific to learned threat. Prior work shows that the PL contributes to behavioral control in other contexts, including innate fear regulation during development and discriminative inhibitory control of cocaine seeking (*57*, *58*). Thus, the present data extend this broader PL function to a learned threat motivational conflict in which reward seeking must be restrained by threat-predictive cues. NAcC manipulation revealed functional specialization along the AP axis. Inactivation of the aNAcC reduced defensive responses and accelerated the approach during conflict, whereas pNAcC inactivation reduced reward pursuit, even in safe trials. This dissociation challenges the view of the NAcC as functionally homogeneous and suggests that the aNAcC acts as a context-sensitive controller for restraint, whereas the pNAcC sustains goal-directed behavior independent of threat (*37*, *59*). Unlike the PL, the aNAcC participates in restraining both learned and innate threats, indicating its broader and more context-independent role.

Projection-specific optogenetics showed that inhibiting PL→aNAcC during conflict reduced defensive responses and shortened decision latency, supporting its causal contribution and mirroring the effects of aNAcC inactivation while sparing innate threat responses. This specificity supports a pathway-level contribution to cue-driven restraint and highlights potential targets for treating disorders characterized by impaired inhibition (*60*). Conversely, activating this pathway disrupted goal-directed behavior even in nonconflict trials, suggesting that, although the PL→aNAcC pathway is necessary for adaptive restraint, excessive drive impairs action selection and underscores the importance of balanced corticostriatal signaling. These results may have translational implications for clinical practice. Pursuing rewards despite remembered threats—a behavior often described as “courage” in ethological and clinical contexts (*51*, *52*)—likely engages overlapping prefrontal-amygdala-striatal networks in humans. Mapping this circuitry provides a framework for studying approach-avoidance conflict across species and for modeling maladaptive decisions, including addiction, where the task could incorporate drug rewards to investigate relapse mechanisms. Because PL terminal photoinhibition was sustained during behavior, these data should be interpreted as pathway-level evidence rather than as proof of a temporally precise synaptic gating mechanism. Future closed-loop experiments recording aNAcC activity during PL terminal inhibition would be needed to define the precise decision-period mechanism.

Although our study establishes the necessity of the PL→aNAcC pathway, further work is needed to fully resolve the temporal and synaptic mechanisms of its control, determine the relevant cell types involved, and define how cortical, hippocampal, and amygdalar inputs precisely influence PL activity during decision-making (*13*, *17*, *21*, *27*, *61*). Dissecting whether this pathway acts via excitation, inhibition, or disinhibition will refine our understanding of how learned threats shape goal-directed actions. Because all experiments were performed in male rats, future studies should test whether the same corticostriatal organization generalizes to females. This is important because recent studies in approach-avoidance and platform-mediated avoidance tasks show that females and males can adopt different behavioral strategies under conflict, including safety-prioritized avoidance, differences in reward seeking under threat, and differences in avoidance extinction or generalization (*62*–*65*). In addition, a recent work links sex differences in contextual fear expression to altered mPFC activity (*66*). These findings make sex an important biological variable for testing whether PL–aNAcC mechanisms operate similarly across sexes. Last, although our seminaturalistic task provides ethological validity, extending analyses to humans through neuroimaging will be critical for assessing the translational relevance of the corticostriatal dynamics described here and for guiding strategies to modulate maladaptive decision-making. Together, these considerations place our findings within a broader framework for understanding adaptive control under motivational conflict.

## MATERIALS AND METHODS

### Animals

Adult male Wistar rats (280 to 300 g; breeding colony at the Institute of Cellular Physiology, UNAM) were housed individually under a 12-hour light/12-hour dark cycle, handled daily, and provided ad libitum water. Rats were food restricted (12 hours/day) on a controlled chow diet with a 5-g weekly bonus to maintain stable motivation for 45 mg of sucrose pellets (Bio-Serv). All experiments were conducted during the light phase (08:00 to 18:00) and were approved by the Institutional Animal Care and Use Committee of the National Autonomous University of Mexico (UNAM; protocol nos. FSB181-22 and FSB211-22) in accordance with national guidelines for the care and use of laboratory animals. Experiments were conducted in male rats to maintain consistency with prior studies and minimize variability related to sex-dependent stress responses.

### Behavioral tasks

All paradigms were adapted from published protocols (*17*, *24*) and are summarized below; the CMC training sequence and cohort-specific testing procedures are schematized in fig. S10.

#### CMC task

The task was conducted in two stainless steel straight alleys (100 cm by 30 cm by 50 cm) inside a sound-attenuating chamber (150 cm by 70 cm by 140 cm). Each alley had two “safe” zones (20 cm by 30 cm; acrylic floor, lever, cue light, speaker, pellet dispenser, and food plate) and a central “danger” zone (60 cm by 30 cm; grid floor delivering scrambled shocks; Coulbourn Instruments). Events (pellet delivery, cues, shocks, and lever presses) were controlled using custom MATLAB scripts. Floors and walls were cleaned between rats with soapy water and 70% ethanol. Rats were first trained to press a lever for 45 mg of sucrose pellets (Bio-Serv) delivered upon cue light presentation. Each session began and ended with a 5-min context-only exposure (no cues or shocks). Conflict training lasted 34 days and comprised three phases: reward, threat, and discrimination.

#### Reward training

Rats confined to a safe zone learned to associate a light cue with the delivery of food. Lever presses during light-on (cue) trials delivered a pellet, whereas presses during light-off (no-cue) trials had no effect on pellet delivery (days 1 to 6). Both light-on and light-off trials were presented simultaneously in the same session and controlled by a custom computer script. Light-on trials ended after a randomly assigned number of rewarded lever presses (5 to 20 presses), whereas light-off trials ended after a randomly assigned duration (30 to 180 s). Once the rats associated the lever with the light-on, they were trained to cross the alley from one safe zone to another in response to the light cue (days 7 to 11, 30 trials per session). Trials ended when the rat crossed and pressed the lever or after 180 s had elapsed without crossing. One to three lever presses were reinforced in the same safe zone per crossing trial to prevent habitual, nonsignaled crossings.

#### Threat training

Although confined to the danger zone, rats received five pairings of white noise (85 dB, 30 s) coterminating with a scrambled foot shock (0.5 mA, 1 s) with variable intertrial intervals (1 to 3 min). The following day, two noise-alone trials were conducted to test threat memory (days 12 to 17). In subsequent sessions, rats were required to cross the alley for a light-cued reward but now under threat: noise-signaled foot shock delivery (0.5 mA) in the danger zone during conflict trials (days 18 to 22). The duration of conflict trials was increased across days to gradually intensify the threat in crossing sessions. Intertrial intervals ranged from 1 to 3 min, and each session was limited to 30 min. Conflict trials lasted 30 s in the first 2 days, 60 s on the third day, 90 s on the fourth day, and 120 s on the fifth day of training. Each trial ended either after the time of crossing elapsed (30 to 120 s) or when rats successfully crossed and pressed the lever in the goal zone.

#### Discrimination training

Short acrylic barriers (9 cm tall) were placed between the safe zones and the grid to define a “choice” zone (Fig. 1A). Each session included three blocks of 10 trials (30 trials) mixing nonconflict (light cue without threat) and conflict (light cue + threat) trials. During conflict trials, the white noise cue remained on from trial onset until the animal crossed to the goal zone or until the 180-s maximum trial duration elapsed. All analyzed conflict test trials involved successful crossing. If a rat did not cross within 180 s, all cues would be turned off, and the latency to cross would be assigned the maximum trial duration of 180 s; the trial would not be counted as a successful crossing. The next trial then began according to the programmed task sequence, without a separate intertrial interval. Conflict trials were presented with a 10% probability during the first three discrimination sessions using a 0.5-mA shock. During the following three sessions, conflict-trial probability was increased to 30% whereas shock intensity was maintained. During the final three discrimination sessions, conflict-trial probability remained at 30%, whereas shock intensity increased by 0.1 mA/day until reaching 0.8 mA. After surgery and recovery, the rats underwent four more sessions, with seven nonconflict and three conflict trials per block, reaching 0.8-mA shock intensity by the last day. Rats that failed to discriminate between trial types during training, or that failed to perform the required reward-directed crossing behavior during the relevant stage, were excluded from the corresponding experiment before final analysis.

#### Conflict test

The conflict test was conducted on the day after the final discrimination session (day 32 for c-Fos experiment or day 35 for remaining experiments). Rats were exposed to 10 crossing trials under the same conditions as the last discrimination session: seven nonconflict trials and three conflict trials (30% probability) presented in random order, with no foot shocks delivered. For optogenetic experiments, the rats completed two consecutive 10-trial blocks (nine nonconflicts and one conflict per block). In the photoinhibition sessions, the laser was activated during one nonconflict and one conflict trial in the first block, starting at trial onset and lasting until the rat crossed (∼90 s) or until 180 s had elapsed. The second block was performed without laser stimulation. In the photoactivation sessions, the first block was unstimulated, and laser stimulation was delivered during the second block following the same protocol. For single-unit recordings, the rats completed six (± 2) consecutive 10-trial blocks (nine nonconflict and one conflict trial each), all without shock delivery. Behavioral performance and neural activity were compared across conflict and nonconflict trials by analyzing the temporal and spatial dynamics of crossing, cue processing, reward-seeking, and threat responses. Given that, during pharmacological inactivation, we observed a decrease in both groups of rats that exhibited extinction behavior after three consecutive conflict trials (figs. S2, B and C, and S5, B and C), we decided to reduce the conflict probability during optogenetic and single-unit recordings. This design minimized extinction while still allowing multiple conflict trials to be collected across the session for neural and behavioral analyses. In addition, for optogenetic experiments, we stimulated only one conflict and one nonconflict trial per block to avoid prolonged laser stimulation, as repeated stimulation across multiple conflict trials could exceed 3 min per trial block depending on crossing duration. Specifically, in photoinhibition experiments, laser stimulation was delivered in the first trial block to assess the immediate effects of inhibition before extinction-related reductions in conflict behavior emerged. In contrast, during photoactivation experiments, stimulation was delivered during the second block because we hypothesized that activation would increase crossing latencies. Delaying stimulation to the second block minimized potential ceiling effects as latencies are typically highest during the initial conflict trial. Cohort-specific final-test structures, including c-Fos mapping, pharmacological inactivation, optogenetic manipulation, and single-unit recordings, are summarized in fig. S10.

### Foraging-mediated innate conflict task

This task was conducted in a custom open field arena (90 cm by 90 cm by 60 cm) placed in a dark room. The arena was divided into a peripheral “safe” zone and a central “threat” zone, brightly illuminated by an overhead light (1500 lux). This illumination enhances natural aversion to open spaces without affecting the periphery. The arena was cleaned using soapy water and 70% ethanol between rats.

#### Conflict test

Separate groups of rats underwent either a conflict or nonconflict version of the task. Under the conflict condition, 30 sucrose pellets (1.35 g in total) were placed in the illuminated center (“threat zone”) to introduce a motivational conflict between innate avoidance and food-seeking behavior. Under the nonconflict condition, no food was placed in the center. To sustain food motivation, 10 sucrose pellets (45 mg each; 0.45 g in total) were placed in each of the four corners of the arena. To reduce neophobia, rats were familiarized with sucrose pellets in their home cages (20 pellets/day for 2 days) before testing. For optogenetic experiments, the laser was alternated ON and OFF throughout the session in 2-min epochs. In all experiments, each rat was individually placed in the “safe” zone and allowed to explore for 10 min. The time spent in the threat and safety zones was recorded. Rats that failed to find and consume pellets in both the corners and the illuminated center were excluded because they did not show evidence of reward-directed engagement with the task. This criterion parallels the CMC exclusion of animals that failed to cross to obtain reward.

### Threat memory retrieval test

Rats were trained and tested in standard conditioning chambers (Coulbourn Instruments, USA). On day 1, they received five auditory fear-conditioning trials in which a tone (75 dB, 30 s) was coterminated with a footshock (0.7 mA, 1 s) delivered through a stainless steel grid floor. On day 2, threat memory was evaluated during two-tone presentations without shock. Each session began with a 5-min acclimation period, followed by tone trials separated by a variable intertrial interval of 120 s. In optogenetic experiments, the laser was activated at tone onset during the first trial and remained on for a 30-s tone duration; the second tone was presented without laser stimulation. Chambers were cleaned between the rats with soapy water and 70% ethanol.

### Reward memory retrieval test

Rats were placed in the safe zone of the CMC apparatus and trained for four consecutive days to press a lever for food pellets (45 mg, dustless precision pellets, Bio-Serv) on a fixed ratio 1 (FR1) schedule. On day 5, reward memory was assessed under the same conditions. In optogenetic experiments, the laser was activated during the first minute of the session and turned off during the second minute. The apparatus was cleaned with soapy water and 70% ethanol between animals.

### Low conflict retrieval test

Rats were trained and tested in standard conditioning chambers (Coulbourn Instruments, USA). During the initial sessions, they learned to press a lever for sucrose pellets (45 mg, dustless precision pellets, Bio-Serv) on a variable interval 60-s (VI-60) schedule. After stable performance, they underwent aversive conditioning with five tone–shock pairings (75 dB tone, 30 s; coterminating with a 0.7-mA, 1-s footshock). The following day, a low-conflict test was conducted with two tones presented without shock while lever pressing remained available.

Each session began with a 5-min baseline and included a variable intertrial interval of 120 s. In the optogenetic experiments, the laser was activated during the first tone (30 s) and remained off during the second tone. Chambers were cleaned between the rats with soapy water and 70% ethanol. During this test, rewards were immediately accessible via lever pressing, and no threat zone navigation was required. Accordingly, this paradigm was operationally defined as a low-conflict condition relative to the CMC task, allowing assessment of threat-induced suppression of instrumental behavior independent of spatial navigation demands.

### Free food intake test

To assess unconditioned feeding, rats were presented in their home cages with 10 g of sucrose pellets (45 mg, dustless precision pellets, Bio-Serv) for 15 min. For optogenetic experiments, 5 g was provided for 4 min, with laser delivery alternated ON and OFF every minute (ON during the first and third minutes; OFF during the second and fourth minutes). The pellet weight was measured before and after the session to calculate the total intake.

### Open field test

Rats were individually placed in a dimly lit open field arena (20 lux) for 10 min. Locomotor activity was quantified as the total distance traveled, and anxiety-like behavior was quantified as the percentage of time spent in the central area. For optogenetic experiments, the laser was alternated ON and OFF in 2-min epochs. The arena was cleaned with soapy water and 70% ethanol between sessions.

### Stereotaxic surgery

Rats were anesthetized with isoflurane (2 to 3% in oxygen) and placed in a stereotaxic frame (Kopf Instruments). For pharmacological inactivation, stainless steel guide cannulae (23-gauge) were bilaterally implanted targeting the PL (+3.0 mm AP, ±0.6 mm ML, −2.5 mm DV), IL (+2.8 mm AP, ±3.1 mm ML, −3.8 mm DV; 30° angle), aNAcC (+2.5 mm AP, ±1.6 mm ML, −5.3 mm DV), or pNAcC (+1.57 mm AP, ±2.0 mm ML, −6.0 mm DV) relative to bregma, with tips positioned 1.0 mm above target. For optogenetics, AAV vectors were infused bilaterally into the PL (+3.2 mm AP, ±0.6 mm ML, −2.5 mm DV from dura) using a glass micropipette connected to a Nanoject-II injector (Drummond; 0.5 μl per site at 110 nl/min) followed by a 10-min diffusion period before retraction; optical fibers [200-μm core, 0.22 numerical aperture (NA); Doric Lenses] were implanted bilaterally over the aNAcC (+2.0 mm AP, ±3.5 mm ML, −6.4 mm DV; 16° angle) and secured with dental acrylic anchored to skull screws. For single-unit recordings, a custom microdrive with a 16-channel array of 35-μm tungsten microwires (California Fine Wire Co.) was unilaterally implanted in the PL; in a subset for optrode validation, Arch-expressing PL neurons were targeted with a fixed 16-wire optrode into the aNAcC (+2.5 mm AP, ±1.6 mm ML, −5.3 mm DV). All rats recovered for at least 7 days before behavioral testing or recording.

### Local drug infusion

To transiently and reversibly inactivate the target regions, a cocktail of γ-aminobutyric acid type A (GABA_A_) and GABA_B_ receptor agonists, muscimol and baclofen (Sigma-Aldrich), was freshly prepared in sterile physiological saline on the day of infusion. Each rat received 0.5 μl per side (250 ng of each compound) delivered 15 min before behavioral testing at a rate of 0.4 μl/min using a programmable microinfusion pump (KD Scientific) connected via polyethylene tubing to a 10-μl Hamilton syringe; injectors were left in place for 2 min postinfusion to facilitate diffusion. Control rats received equivalent saline vehicle infusions, following the same procedure.

### Viral vectors

Viral vectors were obtained from the University of North Carolina Vector Core or Addgene and included AAV5-CaMKII-eArch3.0-eYFP (UNC Vector Core; titer: 3.4 × 10^12^ particles/ml), AAV5-CaMKII-eYFP (UNC Vector Core; titer: 3.6 × 10^12^ particles/ml), and AAV5-CaMKIIa-hChR2 (H134R)-eYFP (Addgene; titer: 2.2 × 10^13^ particles/ml). All vectors were stored at −80°C and used undiluted for microinjections.

### Laser delivery

For optogenetic manipulations, rats expressing the light-driven proton pump archaerhodopsin-3.0 (Arch 3.0) or ChR2 in the PL neuronal bodies received bilateral laser stimulation in the aNAcC. Green diode-pumped solid-state (DPSS) lasers (532 nm, continuous light, 11 mW at fiber tips, adjusted as needed within a 10- to 12-mW range) were used for Arch-mediated photoinhibition, whereas blue DPSS lasers (473 nm, 15 Hz, 5-ms pulse width, 12 mW, adjusted as needed within a 10- to 14-mW range) were used for ChR2-mediated photoactivation, following previously described protocols (*23*). Laser delivery was time locked to specific behavioral events (see Behavioral tasks). Light was transmitted through a laser coupler/shutter (FC/PC; OEM Laser Systems), patch cords (62.5-μm core; Precision Fiber Products), a rotary joint (Doric Lenses), a dual patch cord, and custom-made bilateral optical fibers (assembled in-house from Thorlabs and Precision Fiber Products components). Rats were habituated to the tethering apparatus for 3 days before the first laser session to minimize stress and movement artifacts.

### Multichannel unit recordings and unit analysis

Extracellular recordings from freely behaving rats performing the conflict test were obtained using a custom-fixed 16-channel microwire and an Omniplex acquisition system (Plexon). Custom fixed 16-channel microwire arrays were assembled in the laboratory using a 3D-printed acrylic support. Microwires were aligned through perforated acetate guides and connected to an Omnetics 16-channel EIB with gold pins. One wire was used as the reference or ground connection according to the Omnetics board layout. Signals exceeding the voltage threshold were amplified (×100), digitized at 40 kHz, and stored for offline analysis. Spike sorting was performed with principal components analysis and template matching in an Offline Sorter (Plexon) using a semiautomated pipeline: an initial valley-seeking scan algorithm per channel followed by manual verification. Units were classified as single neurons if they formed well-isolated clusters in the principal component space and exhibited a refractory period of >1 ms; only units stable throughout the session were included. Spike timestamps and behavioral markers were exported to NeuroExplorer (NEX Technologies) for further analysis. Peristimulus time histograms (PSTHs; 300-ms bins) were aligned to the crossing event, defined as the moment the rat’s entire body moved from the choice to the danger zone, using a −20- to 20-s window around the event and averaged across trials.

The −20- to +20-s window was used to visualize broad pericrossing dynamics and classify overall task responsiveness, not as the primary decision-period quantification. This broad window was selected to capture the behavioral sequence surrounding crossing, based on the behavioral profile of electrode-implanted rats shown in Fig. 2I. In those animals, goal zone reward-approach latencies during nonconflict trials were ∼20 s, so the −20- to +20-s window captures the broader pericrossing sequence without being treated as the decision-period quantification. Task-modulated neurons were identified by comparing peri-event (−20 to +20 s) *z*-scores to baseline using a two-tailed Wilcoxon signed-rank test and were classified as conflict-responsive, nonconflict-responsive, responsive in both, or unresponsive. Category distributions were compared using chi-square and pairwise contrasts. Single-neuron heatmaps were plotted using a diverging *z*-score color scale centered at zero, with symmetric limits across negative and positive values.

The main decision-period analysis used a ±600-ms window around crossing onset. A ±300-ms window was used as a stricter peridecision robustness analysis, whereas a ±900-ms window was used to test whether the effect persisted when the analysis extended beyond the immediate crossing period. Decision-period activity was used to classify neurons as excitatory if *z*-score > 2.58 (*P* < 0.01, two-tailed) or inhibitory if *z*-score < −1.96 (*P* < 0.05, two-tailed), when their activity crossed the predefined *z*-score threshold in at least one bin within the ±600-ms pericrossing window. These criteria refined the inclusion criteria for peri-event comparisons and polarity assessments. Significant neurons were displayed in heatmaps generated from *z*-scored firing activity aligned to crossing onset. Neurons were separated according to response polarity (excitatory: conflict *n* = 13, nonconflict *n* = 12; inhibitory: conflict *n* = 2, nonconflict *n* = 30) and sorted on the basis of their mean pericrossing activity within a −600- to +600-ms window around the event. For visualization purposes, plots were restricted to a −10- to 10-s window, although analyses were performed using the full −20- to +20-s peri-event window. Population-level comparisons between conflict- and nonconflict-responsive neurons used two-tailed unpaired *t* tests on mean *z*-scores in the ±600-ms window. The response magnitude was quantified as the AUC for the same window, and the same proportions of significantly excited or inhibited neurons under each condition were derived from AUC-based classifications. To minimize the visual influence of extreme *z*-score values, the heatmap color scaling was capped at the 99th percentile of the *z*-score distribution. The distribution of recorded units across animals was examined for both the broad task-responsiveness analysis and the signed pericrossing analysis to ensure that the reported effects were not attributable to a single animal.

### Optrode recordings

To validate the optogenetic modulation of PL terminals in the aNAcC, rats expressing Arch in the PL were implanted with optrode arrays in the aNAcC 4 weeks after viral delivery. Each optrode consisted of 16 tungsten microwires (35 μm in diameter; California Fine Wire Company) arranged around a central optical fiber (200-μm core, 0.22 NA). Single units were recorded while applying a repeated laser-pulse protocol of 10 alternating 20-s epochs: 10 s of continuous green laser photoinhibition (532 nm, ∼10 to 12 mW at the fiber tip) followed by 10 s without light. The 10-s stimulation duration was chosen because it corresponded to the shortest stimulation epochs used during behavior. Establishing that this minimal duration was sufficient to modulate neural activity validated that the longer stimulation periods used during behavioral testing would also effectively alter neural activity. Spiking data were acquired and stored for offline spike sorting (Offline Sorter, Plexon) and spike train analysis (NeuroExplorer, NEX Technologies). Firing rate changes were assessed by comparing spike counts during laser ON versus OFF epochs using Wilcoxon signed-rank tests (1-s bins). For each 1-s bin, *z*-scores were calculated relative to baseline activity during the preceding 10-s OFF period, with units classified as excited if *z* > 2.58 (*P* < 0.01) or inhibited if *z* < −1.96 (*P* < 0.05) during laser activation.

### Histology

At the conclusion of behavioral experiments, rats were deeply anesthetized with sodium pentobarbital (150 mg/kg, intraperitoneally) and transcardially perfused with 0.9% saline followed by 4% paraformaldehyde (PFA) in phosphate-buffered saline (PBS). Brains were extracted, postfixed overnight in 4% PFA at 4°C, and cryoprotected in 30% sucrose in PBS until saturated. Coronal sections (50 μm) were sliced using a cryostat (Leica CM1520) and processed for Nissl staining. Sections were examined under a bright-field microscope (Nikon H550S) to verify the cannula tip locations, electrode array placements, and viral expression sites. Only animals with confirmed placements within the intended target structures were included in the analyses.

### Immunohistochemistry

Rats were perfused with 0.9% saline followed by 4% PFA 90 min after the conflict or nonconflict test sessions. Brains were postfixed overnight in PFA at 4°C and then cryoprotected in 30% sucrose in PBS. Coronal sections (40 μm) were sliced using a cryostat (Leica CM1520) at multiple rostrocaudal levels of the mPFC and nucleus accumbens. For c-Fos immunostaining, the sections were rinsed six times in tris-buffered saline (TBS) with 0.1% Tween 20, incubated in 3% hydrogen peroxide (Sigma-Aldrich) for 10 min to quench endogenous peroxidase activity, and blocked for 2 hours at room temperature in 4% normal goat serum (Vector Laboratories), 4% bovine serum albumin (Santa Cruz Biotechnology), and 0.1% Triton X-100 in TBS-Tween. Sections were then incubated for 48 hours at room temperature with rabbit polyclonal anti-c-Fos (1:1000; Millipore), followed by a 1-hour incubation with biotinylated goat anti-rabbit secondary antibody (1:500; Jackson ImmunoResearch). The signal was amplified using the ABC Elite Kit (Vector Laboratories) and visualized using DAB (SK-4100; Vector Laboratories), after which the tissues were washed three times in PBS. For NeuN immunostaining, the same protocol was applied using rabbit anti-NeuN conjugated with Alexa Fluor 647 (1:2000; Abcam). Sections were mounted on gelatin-coated slides and coverslipped with aqueous mounting medium.

### Microscopy and image analysis

For bright-field microscopy, c-Fos-labeled neurons were quantified automatically using ImageJ (RRID: SCR_003070). Sections were imaged at 10× magnification with a Nikon H550S microscope equipped with a digital camera, acquiring micrographs at three AP levels from bregma for each region of interest: mPFC (+3.5 to +2.5 mm) and nucleus accumbens (+2.5 to +1.5 mm). Neurons were classified as c-Fos positive if the nucleus was clearly distinguishable from the background, with an area of 25 to 250 μm^2^ and circularity of ≥0.60. A consistent, predetermined intensity threshold was applied across all samples using ImageJ’s automated particle analysis function to ensure objective identification of positive nuclei. For each region, the labeled cells were counted in three sections per rat from one hemisphere and averaged. For optogenetic experiments, fluorescence images of NeuN-immunostained tissues were acquired using a confocal/two-photon microscope (Zeiss LSM710) at 10×, 20×, and 40× magnification. *Z*-stacks of 18 optical sections were collected at 3-μm intervals, spanning ∼50 μm of tissue per region; maximum intensity projections were generated along the *z* axis, and channels were merged. Representative images of viral expression were obtained using TILE scans at 10× or 20× magnification, with a pinhole of ∼10 μm. All images were processed using ImageJ, pseudocolored according to the fluorophore, and adjusted for brightness and contrast.

### Data collection and analysis

Initial sample sizes, final analyzed sample sizes, and reasons for exclusion for each experiment are provided in table S1. Exclusions were based on predefined criteria, including failure to meet behavioral inclusion criteria, cannula or fiber/virus misplacement, injector blockage, implant detachment, absence of detectable single-unit activity, or health-related complications after surgery. All behaviors were recorded using overhead digital video cameras (Provision D-380D5) positioned above each apparatus and analyzed using ANY-maze 7.5, an automated tracking software (Stoelting, USA). The straight alley was divided into four zones (start: 19 cm; choice: 10 cm; danger: 42 cm; goal: 29 cm) to assess temporal and spatial behavioral patterns, including cue responses, crossings, reward-seeking, and defensive reactions. For occupancy analyses, start, danger, and goal zones were treated as mutually exclusive spatial compartments using the body-center tracking point. The choice zone was analyzed separately as a decision-related region located at the boundary between the start/safe and danger zones and was defined using the head-tracking point. Therefore, plotted choice zone values should not be interpreted as one component of the same mutually exclusive 100% occupancy budget used for start, danger, and goal zones. Tracking at three body points (head, center, and tail) provided data on time spent and entries per zone; the center point determined presence in the start, danger, and goal zones, whereas the head point determined the choice zone presence (within 10 mm for a successful visit). The percentage time per zone was calculated as: (time per zone × 100)/total time in the start, danger, and goal zones. Additional measures included freezing bouts (≥1000-ms immobility in the start zone), risk-assessment events (choice zone entries with approach/avoidance oscillations toward the reward site), crossing speed (m/s across the danger zone), and latency to press in the goal zone (sum of times in start, danger, and goal zones). Movement heatmaps were generated from head-tracking data, with a 10-s maximum for the hottest color in the CMC task and a 60-s maximum in the foraging-mediated conflict task. In all tests, freezing bouts were defined in the main analyses using a 1-s minimum immobility criterion. To determine whether the findings depended on this threshold, we repeated the key analyses using 300-, 500-, and 750-ms criteria, which are reported in fig. S11 as robustness analyses. Distance traveled and center time in the open field were automatically computed in the ANY-maze. Food intake was calculated as follows: (food consumed × 100)/food offered, with pellet weights measured (each pellet weighed 45 mg) before and 30 min after access; intake was manually recorded by experimenters blinded to the condition. Data were analyzed using unpaired/paired two-tailed *t* tests and mixed-design two-way analyses of variance (ANOVAs), followed by Bonferroni when significant trial × group interactions were found. To determine whether behavioral effects were influenced by the order of conflict-trial presentation, behavioral measures were analyzed using GEE models implemented in Python with the statsmodels package. Trial number, group, and their interaction were included as fixed effects, whereas rat identity was included as the clustering variable to account for repeated observations within subjects. For cluster and dendrogram analysis, behavioral latency measures obtained during nonconflict and conflict conditions were analyzed using hierarchical agglomerative clustering in Python (scikit-learn). Numerical values from each subject were grouped using Ward’s linkage method with Euclidean distance as the similarity metric. The number of clusters was set to k = 2 in the agglomerative clustering algorithm. To visualize hierarchical relationships among subjects, a dendrogram was generated using SciPy’s Ward linkage. Analyses were performed using GraphPad Prism 10 and the Python statsmodels package, including effect size calculations, with statistical significance set at *P* < 0.05.
