# Supplementary material for: Top-down corticostriatal control of adaptive restraint during motivational conflict
Source: Sci Adv. 2026 Sep 11;12(37):eaeg2327. doi: 10.1126/sciadv.aeg2327 (PMC13564925; doi:10.1126/sciadv.aeg2327)
Supplement: Supplementary file 1 [file sciadv.aeg2327_sm.pdf]

Science Advances
AAAS

Supplementary Materials for

Top-down corticostriatal control of adaptive restraint during motivational conflict

Elizabeth Illescas-Huerta *et al.*

Corresponding author: Elizabeth Illescas-Huerta, e.illescashuerta@ufl.edu;
Francisco Sotres-Bayon, sotres@ifc.unam.mx

Sci. Adv. **12**, eaeg2327 (2026)
DOI: 10.1126/sciadv.aeg2327

The PDF file includes:

Figs. S1 to S11
Legend for movie S1
Table S1

Other Supplementary Material for this manuscript includes the following:

Movie S1

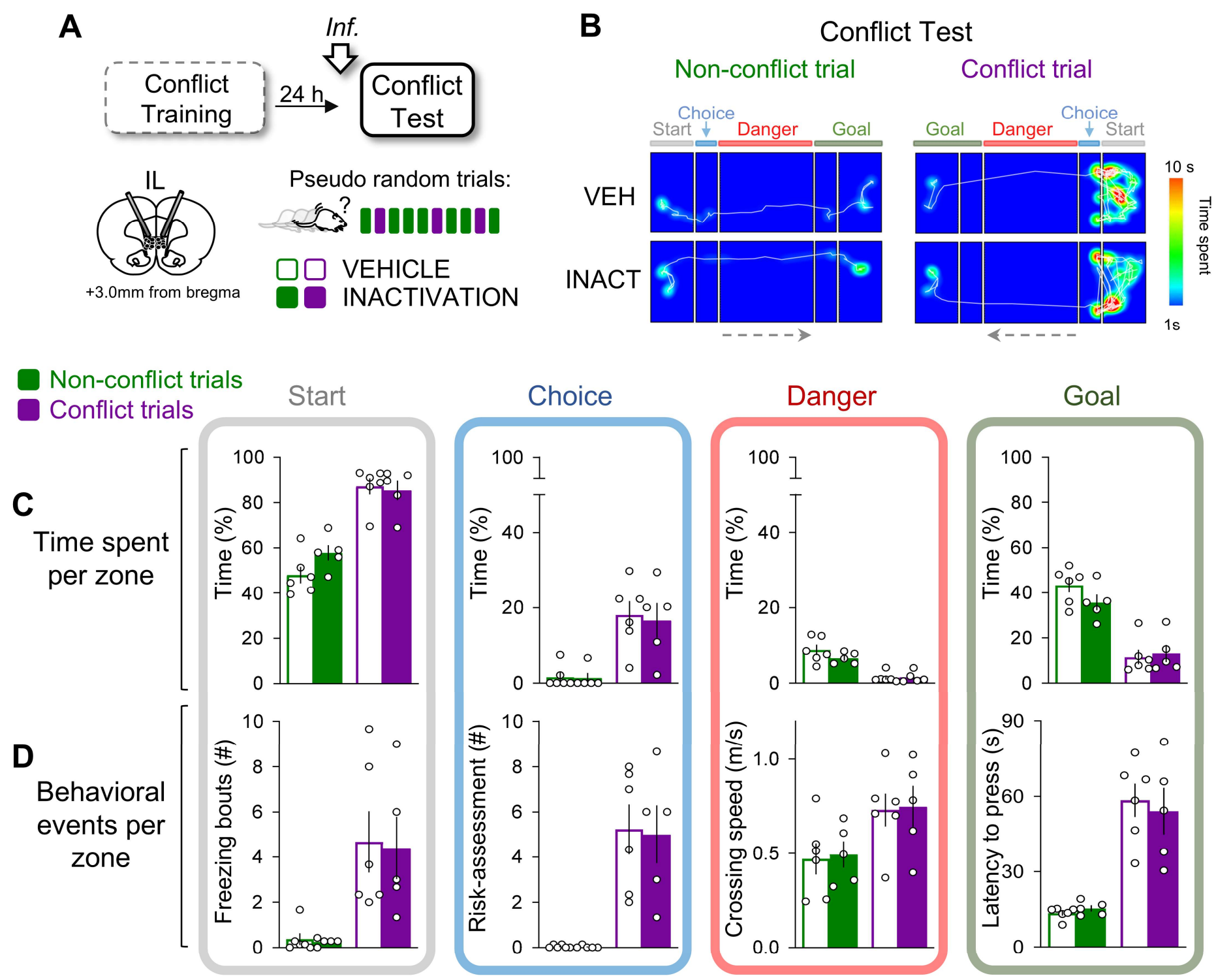

Fig. S1. IL inactivation does not affect restraint under learned threat.

(**A**) Bilateral cannula placements in the infralimbic cortex (IL). Rats received vehicle (VEH, n = 6) or muscimol + baclofen (INACT, n = 5) before the conflict test.

(**B**) Representative tracks and heat maps during conflict (reward + threat) and non-conflict (reward-only) trials.

(**C**) IL inactivation did not significantly change time in the start (trials, $F(1, 9) = 65.87$, $P < 0.0001$, $\eta p^2 = 0.87$; group, $F(1, 9) = 1.31$, $P = 0.28$, $\eta p^2 = 0.12$; trial × group interaction, $F(1, 9) = 1.98$, $P = 0.19$, $\eta p^2 = 0.18$), choice (trials, $F(1, 9) = 24.35$, $P = 0.0008$, $\eta p^2 = 0.73$; group, $F(1, 9) = 0.09$, $P = 0.76$, $\eta p^2 = 0.01$; trial × group interaction, $F(1, 9) = 0.03$, $P = 0.85$, $\eta p^2 = 0.004$), danger (trials, $F(1, 9) = 61.55$, $P < 0.0001$, $\eta p^2 = 0.87$; group, $F(1, 9) = 0.96$, $P = 0.35$, $\eta p^2 = 0.09$; trial × group interaction, $F(1, 9) = 2.15$, $P = 0.17$, $\eta p^2 = 0.19$), or goal zones (trials, $F(1, 9) = 51.37$, $P < 0.0001$, $\eta p^2 = 0.85$; group, $F(1, 9) = 1.04$, $P = 0.33$, $\eta p^2 = 0.02$; trial × group interaction, $F(1, 9) = 1.48$, $P = 0.25$, $\eta p^2 = 0.14$) across trial types.

(**D**) Defensive behaviors—freezing (trials, $F(1, 9) = 17.25$, $P = 0.0025$, $\eta p^2 = 0.65$; group, $F(1, 9) = 0.04$, $P = 0.84$, $\eta p^2 = 0.004$; trial × group interaction, $F(1, 9) = 0.005$, $P = 0.94$, $\eta p^2 = 0.0006$), risk assessment (trials, $F(1, 9) = 36.19$, $P = 0.0002$, $\eta p^2 = 0.80$; group, $F(1, 9) = 0.02$, $P = 0.88$, $\eta p^2 = 0.002$; trial × group interaction, $F(1, 9) = 0.01$, $P = 0.90$, $\eta p^2 = 0.001$), velocity (trials, $F(1, 9) = 11.54$, $P = 0.0079$, $\eta p^2 = 0.56$; group, $F(1, 9) = 0.04$, $P = 0.84$, $\eta p^2 = 0.004$; trial × group interaction, $F(1, 9) = 0.001$, $P = 0.96$, $\eta p^2 = 0.0002$) and press latency (trials, $F(1, 9) = 54.00$, $P < 0.0001$, $\eta p^2 = 0.85$; group, $F(1, 9) = 0.05$, $P = 0.82$, $\eta p^2 = 0.005$; trial × group interaction, $F(1, 9) = 0.30$, $P = 0.59$, $\eta p^2 = 0.03$) were unaffected by IL inactivation in both trial types. Zone definitions are described in Methods.

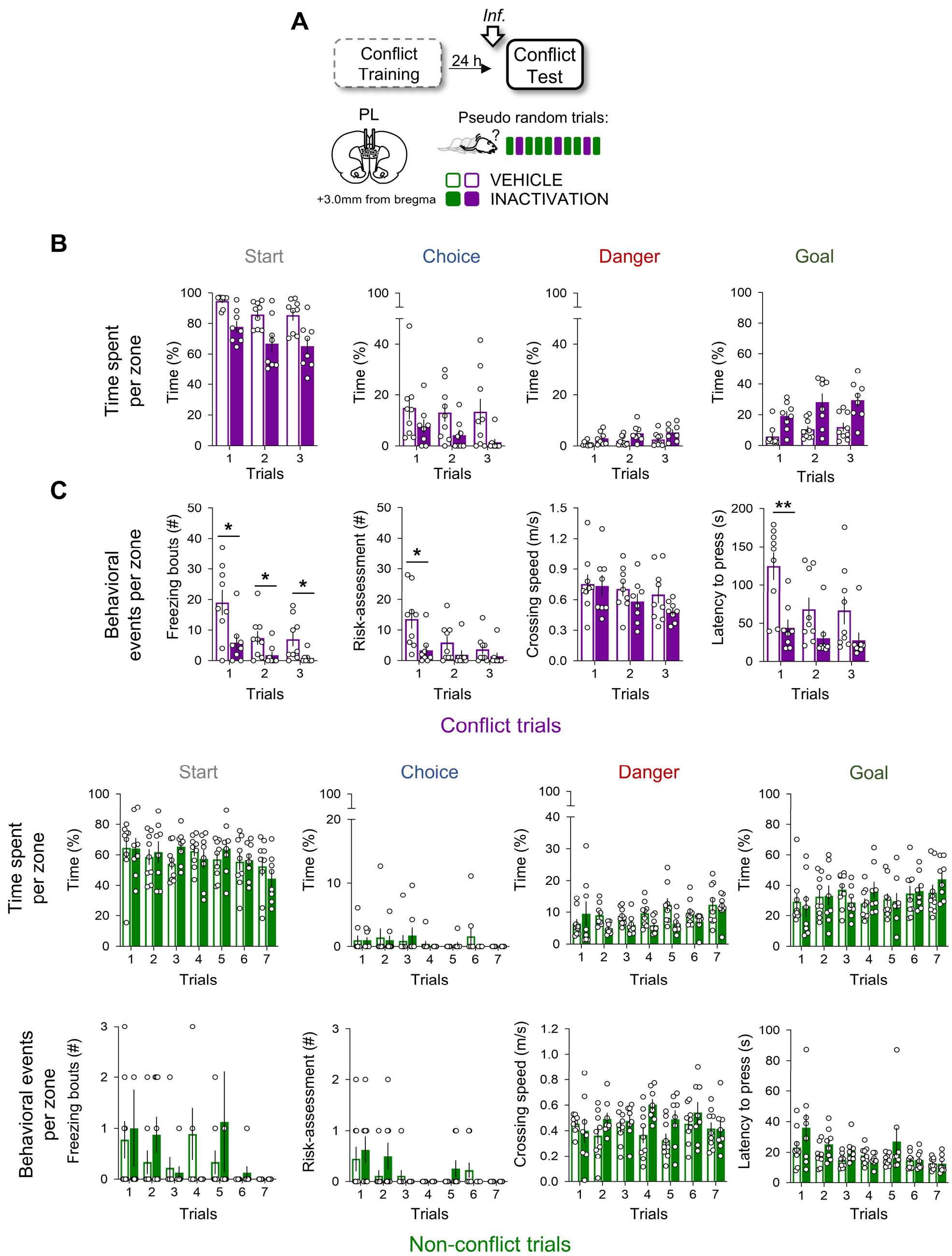

Figure S2. Trial-by-trial analysis during conflict and non-conflict behavior following PL inactivation.

(**A**) Cannula placement in PL. Before testing, rats received PL infusions of saline (VEH, n = 9) or muscimol+baclofen (INACT, n = 8).
(**B**) PL inactivation did not change the time spent in the start (trials, $F(1.73, 26.02) = 12.41$, $P = 0.0003$, $\eta p^2 = 0.45$; group, $F(1, 15) = 14.79$, $P = 0.0016$, $\eta p^2 = 0.49$; trial × group interaction, $F(2, 30) = 0.24$, $P = 0.78$, $\eta p^2 = 0.01$), choice (trials, $F(1.77, 25.76) = 0.71$, $P = 0.47$, $\eta p^2 = 0.045$;

group, $F(1, 15) = 7.56$, $P = 0.014$, $\eta p^2 = 0.33$; trial × group interaction, $F(2, 30) = 0.26$, $P = 0.77$, $\eta p^2 = 0.01$), danger (trials, $F(15.09, 22.64) = 10.55$, $P = 0.001$, $\eta p^2 = 0.41$; group, $F(1, 15) = 5.93$, $P = 0.027$, $\eta p^2 = 0.28$; trial × group interaction, $F(2, 30) = 0.41$, $P = 0.66$, $\eta p^2 = 0.02$), and goal zones (trials, $F(1.86, 28.03) = 7.064$, $P = 0.039$, $\eta p^2 = 0.32$; group, $F(1, 15) = 14.91$, $P = 0.0001$, $\eta p^2 = 0.49$; trial × group interaction, $F(2, 30) = 0.52$, $P = 0.59$, $\eta p^2 = 0.03$) across individual conflict trials.

(**C**) PL inactivation decreased freezing (trials, $F(1.37, 20.55) = 17.85$, $P = 0.0001$, $\eta p^2 = 0.54$; group, $F(1, 15) = 7.42$, $P = 0.016$, $\eta p^2 = 0.33$; trial × group interaction, $F(2, 30) = 3.47$, $P = 0.044$, $\eta p^2 = 0.18$; Trial 1: $P = 0.017$, 95% CI [2.70, 23.54], Cohen's $d = 1.32$; Trial 2: $P = 0.044$, 95% CI [0.17, 11.22], Cohen's $d = 1.09$; Trial 3: $P = 0.029$, 95% CI [0.77, 11.48], Cohen's $d = 1.25$), risk assessments (trials, $F(1.72, 25.80) = 11.27$, $P = 0.0005$, $\eta p^2 = 0.42$; group, $F(1, 15) = 5.21$, $P = 0.03$, $\eta p^2 = 0.25$; trial × group interaction, $F(2, 30) = 4.99$, $P = 0.013$, $\eta p^2 = 0.24$; Trial 1: $P = 0.014$, 95% CI [2.43, 17.93], Cohen's $d = 1.38$), and latency to press in the goal zone (trials, $F(1.85, 27.80) = 10.70$, $P = 0.0005$, $\eta p^2 = 0.41$; group, $F(1, 15) = 8.73$, $P = 0.009$, $\eta p^2 = 0.36$; trial × group interaction, $F(2, 30) = 3.64$, $P = 0.038$, $\eta p^2 = 0.19$; Trial 1: $P = 0.002$, 95% CI [35.35, 125.4], Cohen's $d = 1.87$) across individual conflict trials.

(**D**) PL inactivation did not change the time spent in any of the evaluated zones across individual non-conflict trials.

(**E**) PL inactivation did not change freezing, risk assessments, speed, and latency to press across individual non-conflict trials.

Data are mean ± SEM; $*P < 0.05$, $**P < 0.01$, Bonferroni post hoc test following one-way mixed-design ANOVA or Student's t-test. The empty circles represent individual rats, and effect sizes are reported as ηp^2 and Cohen's d.

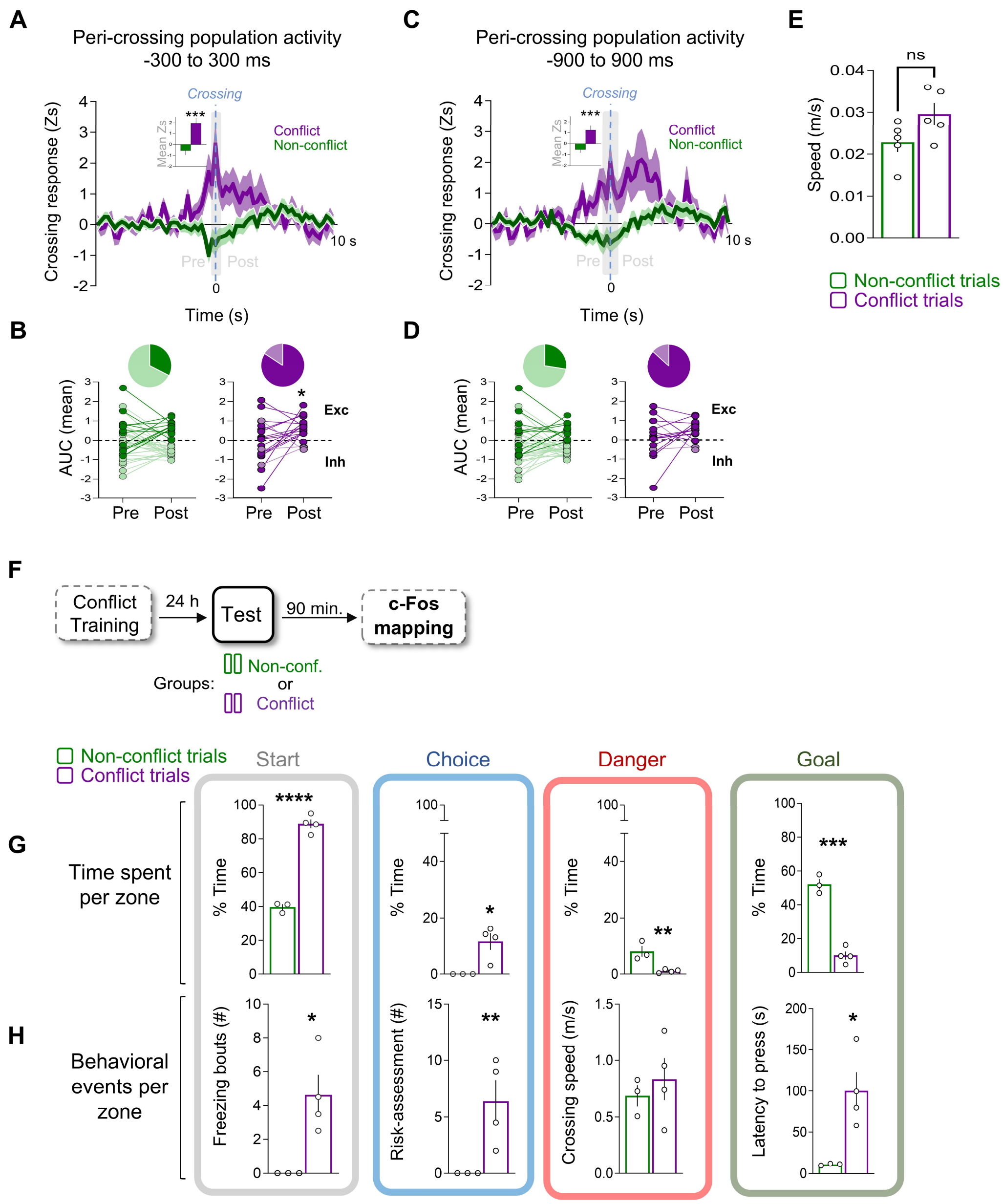

Figure S3. PL neuronal dynamics across short and longer temporal windows and full behavioral profiling in c-Fos rats.

(**A**) Average normalized firing rates (z-scores) of conflict- and non-conflict-responsive neurons in a shorter peri-crossing time window (-300 to 300ms). Conflict neurons (n = 19) showed ramping, a peri-entry peak, and rapid baseline return, whereas non-conflict neurons (n = 40) displayed slower, tonic patterns. Inset: mean activity (±300 ms) was higher in conflict than in non-conflict neurons ($t(57) = 3.95$, $P = 0.0002$, 95% CI [-3.817, -1.251], Cohen's $d = 1.10$). (**B**) Area under the curve confirmed excitation in conflict-excited neurons vs. baseline ($t(18) = -2.25$, $P = 0.037$, 95% CI [-1.260, -0.04249], Cohen's $d = 0.51$), but no modulation was observed

in non-conflict neurons (t(39) = 0.78, P = 0.43, 95% CI [-0.2063, 0.4670], Cohen's *d* = 0.12). Pie charts show excitation/inhibition in conflict (84%/16%) and non-conflict (33%/67%).
(**C**) Average normalized firing rates (z-scores) of conflict- and non-conflict-responsive neurons in a longer peri-crossing time window (-900 to 900 ms). Conflict neurons (n = 15) showed ramping, a shorter peri-entry peak, and rapid baseline return with a second sustained increase in activity. Non-conflict neurons (n = 40) showed minimal modulation. Inset: mean activity (±900 ms) was higher in conflict than in non-conflict neurons (t(53) = 3.53, P = 0.0009, 95% CI [-2.817, -0.7767], Cohen's *d* = 1.06).
(**D**) Area under the curve did not show significant excitation in conflict-excited neurons vs. baseline (t(14) = 1.15, P = 0.26), and modulation in non-conflict neurons (t(39) = 0.26, P = 0.79). Pie charts show excitation/inhibition in conflict (86%/14%) and non-conflict (27%/73%).
(**E**) Movement speed within the safe zone was similar between conflict and non-conflict trials across recorded rats (t(4) = 1.48, P = 0.21).
(**F**) Rats (non-conflict, n = 3; conflict, n = 4) were perfused 90 min after the test for c-Fos mapping.
(**G**) Conflict rats spent more time in the start (t(5) = 14.55, P < 0.0001, 95% CI [-57.74, -40.40], Cohen's d = 11.11) and choice zones (t(5) = 3.35, P = 0.02, 95% CI [-20.53, -2.73], Cohen's d = 2.56), but less time in the danger (t(5) = 4.36, P = 0.007, 95% CI [2.92, 11.26], Cohen's d = 3.33) and goal zones (t(5) = 10.75, P = 0.0001, 95% CI [31.95, 52.03], Cohen's d = 8.21) than non-conflict rats.
(H) Conflict rats increased freezing (t(5) = 3.26, P = 0.02, 95% CI [-8.265, -0.9849], Cohen's *d* = 2.49), risk assessment (t(5) = 2.86, P = 0.03, 95% CI [-12.11, -0.6384], Cohen's *d* = 2.18) and crossing latency to press (t(5) = 3.32, P = 0.02, 95% CI [-158.2, -20.24], Cohen's *d* = 2.53). In contrast, no differences in the crossing speed were found between conflict and non-conflict rats (t(5) = 0.63, P = 0.55).
Zone definitions are described in Methods. Data are mean ± SEM; *P < 0.05, **P < 0.01, ***P < 0.001, ****P < 0.0001, Student's t-test. The empty circles represent individual rats, and effect sizes are reported as Cohen's *d*.

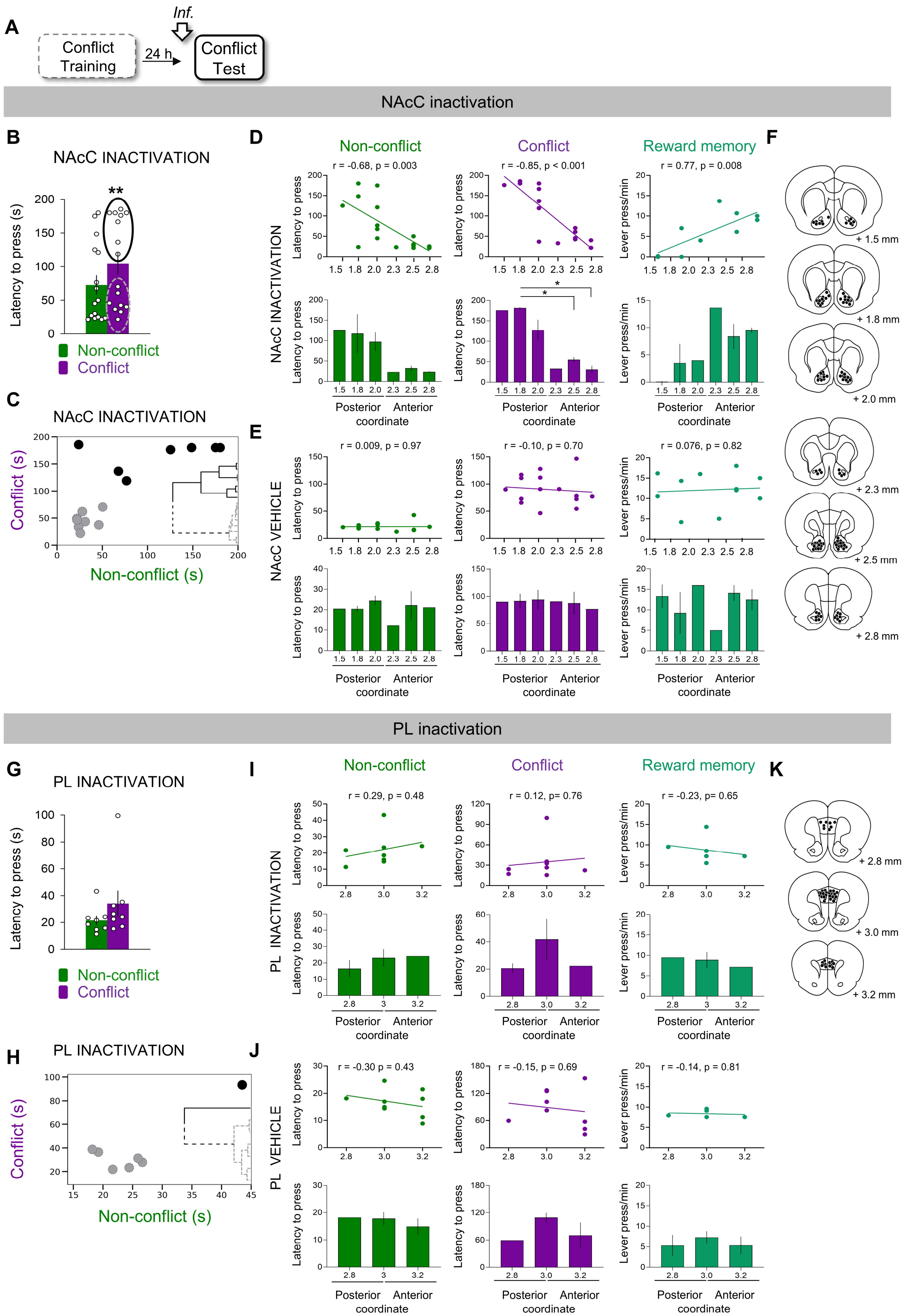

A
Inf.
Conflict Training
24 h
Conflict Test
NAcC inactivation
B
NAcC INACTIVATION
**
Latency to press (s)
Non-conflict
Conflict
C
NAcC INACTIVATION
Conflict (s)
Non-conflict (s)
D
Non-conflict
Conflict
Reward memory
r = -0.68, p = 0.003
r = -0.85, p < 0.001
r = 0.77, p = 0.008
NAcC INACTIVATION
Latency to press
Lever press/min
Posterior Anterior coordinate
E
NAcC VEHICLE
r = 0.009, p = 0.97
r = -0.10, p = 0.70
r = 0.076, p = 0.82
F
+ 1.5 mm
+ 1.8 mm
+ 2.0 mm
+ 2.3 mm
+ 2.5 mm
+ 2.8 mm
PL inactivation
G
PL INACTIVATION
Latency to press (s)
Non-conflict
Conflict
H
PL INACTIVATION
Conflict (s)
Non-conflict (s)
I
Non-conflict
Conflict
Reward memory
r = 0.29, p = 0.48
r = 0.12, p= 0.76
r = -0.23, p= 0.65
PL INACTIVATION
J
PL VEHICLE
r = -0.30 p = 0.43
r = -0.15, p = 0.69
r = -0.14, p = 0.81
Latency to press
Lever press/min
Posterior Anterior coordinate
K
+ 2.8 mm
+ 3.0 mm
+ 3.2 mm

Fig. S4. Clustering and anterior-posterior analysis of NAcC inactivation effects with PL comparison.

NAcC placements were first classified using the clustering procedure described in the Methods section and then analyzed as a function of the AP coordinate to relate behavioral effects to anatomical position. PL placements were included as a comparison region to test whether AP-coordinate relationships reflected generic placement variability or a NAcC-specific rostrocaudal organization.
(**A**) Experimental timeline for NAcC (n = 16) and PL (n = 8) inactivation before the conflict test.
(**B**) NAcC inactivation produced heterogeneous behavioral effects, with significant differences between conflict and non-conflict latencies ($t(15) = 3.15$, $P = 0.006$, 95% CI [-53.70, -10.35], Cohen's $d = 0.79$).
(**C**) Unsupervised clustering revealed two NAcC subgroups (gray vs. black) corresponding to anterior (+2.8 to +2.3 mm) and posterior (+2.0 to +1.5 mm) infusion sites.
(**D**) Left-middle, scatterplots showing the relationship between cannula anteroposterior (AP) coordinates of NAcC and latency to press during non-conflict and conflict trials in the inactivation group. More anterior NAcC inactivation sites (+2.3mm, +2.5mm, +2.8mm) were correlated with shorter latencies, whereas more posterior sites (+1.5mm, +1.8mm, +2.0mm) were associated with longer latencies.
Right, scatterplots showing the relationship between cannula anteroposterior (AP) coordinates and lever press per minute during the reward memory test. More anterior NAcC inactivation sites (+2.3mm, +2.5mm, +2.8mm) were associated with higher rates of lever pressing, whereas more posterior sites (+1.5mm, +1.8mm, +2.0mm) were associated with lower lever presses. Bottom, latency to press and lever presses grouped by AP coordinates. Inactivation of NAcC posterior produced a greater increase in latency to press during conflict trials ($F(5, 10) = 7.71$, $P = 0.003$, $\eta p^2 = 0.79$; Coordinate +1.8 vs +2.5: $P = 0.015$, 95% CI [20.11, 233.9], Cohen's $d = 3.47$; Coordinate +1.8 vs +2.8: $P = 0.016$, 95% CI [23.09, 278.6], Cohen's $d = 4.12$), whereas non-conflict ($F(5, 10) = 2.05$, $P = 0.15$), and lever pressing for rewards conflict ($F(5, 4) = 4.08$, $P = 0.09$) showed non-significant tendencies to differ between coordinates.
(**E**) Scatterplots showing no correlation between cannula anteroposterior (AP) coordinates of NAcC and latency to press in both type trials and reward memory in the vehicle group. Mean latency to press and lever presses grouped by AP coordinates showed no significant differences in non-conflict ($F(5, 9) = 0.352$, $P = 0.86$), conflict trials ($F(5, 9) = 0.045$, $P = 0.99$), and reward memory ($F(5, 5) = 0.94$, $P = 0.52$).
(**F**) Schematic representation of cannula placements across the rostrocaudal extent of the NAcC. Black circles indicate injection sites located at AP coordinates +2.3mm, +2.5mm, +2.8mm, +1.5mm, +1.8mm, +2.0mm relative to bregma.
(**G**) PL inactivation produced homogeneous behavioral effects between conflict and non-conflict latencies ($t(7) = 1.74$, $P = 0.12$).
(**H**) Unsupervised clustering revealed less-defined separation among observations, with no clearly distinguishable clusters.
(**I**) Scatterplots showing no correlation between cannula anteroposterior (AP) coordinates of PL (anterior, +3.2 mm; middle, +3.0 mm; posterior, +2.8 mm) and latency to press in both type trials and reward memory during the inactivation group. Latency to press and lever presses grouped by AP coordinates of PL showed non-significant differences in non-conflict ($F(2, 5) = 0.29$, $P = 0.75$), conflict trials ($F(2, 5) = 0.45$, $P = 0.66$), and reward memory ($F(2, 3) = 0.10$, $P = 0.90$).

(**J**) Scatterplots showing no correlation between cannula anteroposterior (AP) coordinates of PL (anterior, +3.2mm; middle, +3.0 mm; posterior, +2.8 mm) and latency to press in both type trials and reward memory in the vehicle group. Mean latency to press and lever presses grouped by AP coordinates of PL showed no significant differences in non-conflict ($F(2, 6) = 0.35$, $P = 0.71$), conflict trials ($F(2, 6) = 1.06$, $P = 0.40$), and reward memory ($F(2, 5) = 0.38$, $P = 0.69$).
(**K**) Schematic representation of cannula placements across the rostrocaudal extent of the PL. Black circles indicate injection sites located at AP coordinates +2.8 mm, +3.0 mm, and +3.2 mm relative to bregma.
Data are mean ± SEM; $^{*}P < 0.05$, $^{**}P < 0.01$, Bonferroni post hoc test following one-way ANOVA or Student's t-test. The empty circles represent individual rats, and effect sizes are reported as ηp^2 and Cohen's *d*.

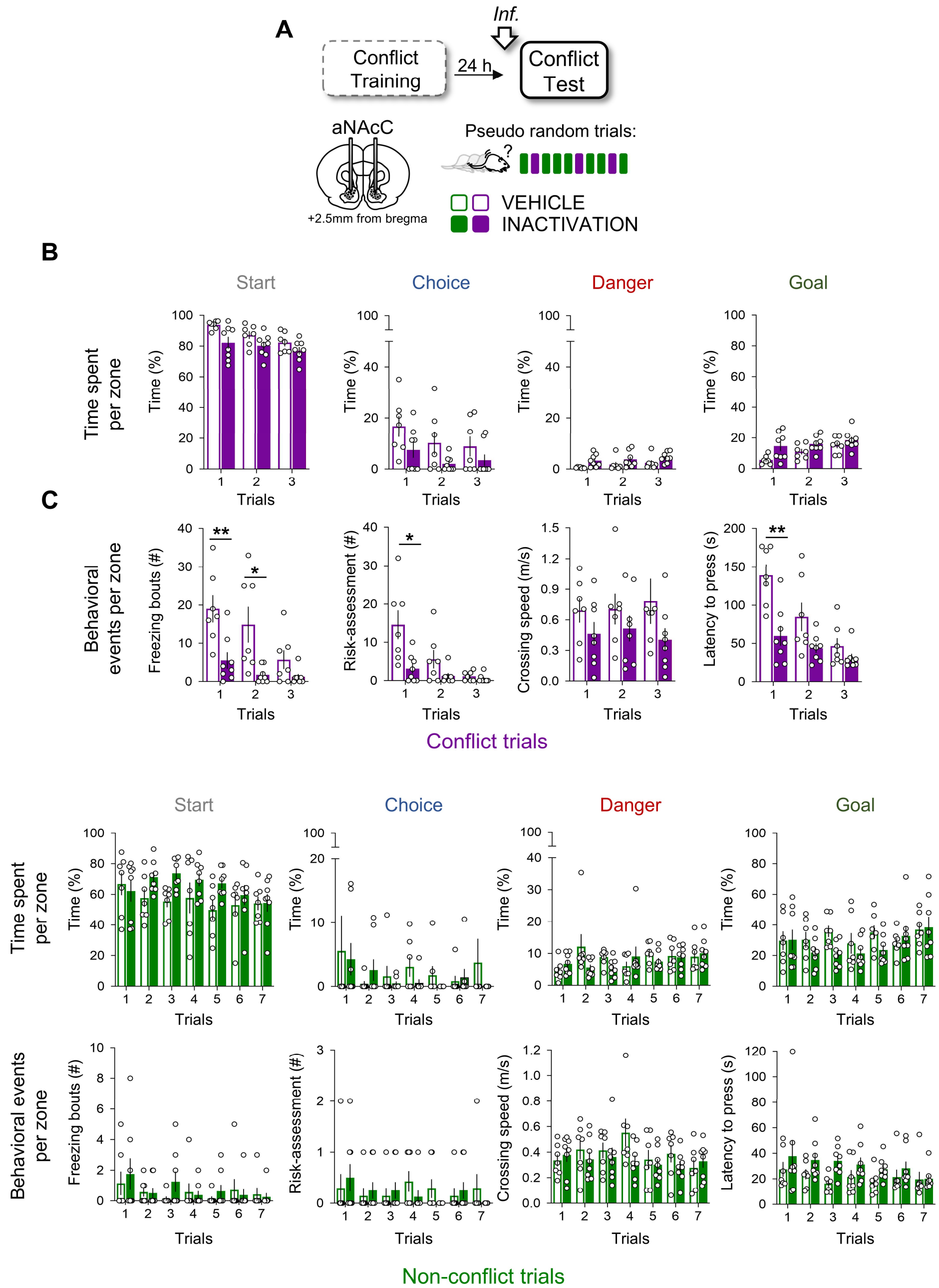

Figure S5. Trial-by-trial analysis during conflict and non-conflict behavior following aNAcC inactivation.

(**A**) Cannula placements in anterior NAcC (aNAcC). Rats received vehicle (VEH, n = 7) or muscimol+baclofen (INACT, n = 8) before the conflict test.
(**B**) aNAcC inactivation did not change the time spent in the start (trials, $F(1.74, 22.64) = 8.27$, $P = 0.002$, $\eta p^2 = 0.38$; group, $F(1, 13) = 7.25$, $P = 0.01$, $\eta p^2 = 0.35$; trial × group interaction, F(2,

26) = 1.30, P = 0.28, ηp^2 = 0.09), choice (trials, F(1.95, 25.37) = 2.89, P = 0.07, ηp^2 = 0.18; group, F(1, 13) = 5.82, P = 0.03, ηp^2 = 0.30; trial × group interaction, F(2, 26) = 0.24, P = 0.78, ηp^2 = 0.01), danger (trials, F(1.60, 20.84) = 4.01, P = 0.04, ηp^2 = 0.23; group, F(1, 13) = 4.85, P = 0.046, ηp^2 = 0.27; trial × group interaction, F(2, 26) = 0.30, P = 0.74, ηp^2 = 0.027), and goal zones (trials, F(1.85, 24.15) = 7.84, P = 0.002, ηp^2 = 0.37; group, F(1, 13) = 6.0, P = 0.029, ηp^2 = 0.31; trial × group interaction, F(2, 26) = 1.44, P = 0.25, ηp^2 = 0.09) across individual conflict trials.

(**C**) aNAcC inactivation decreased freezing (trial, F(1.19, 15.49) = 10.99, P = 0.003, ηp^2 = 0.45; group, F(1, 13) = 11.67, P = 0.004, ηp^2 = 0.47; trial × group interaction, F(2, 26) = 3.71, P = 0.038, ηp^2 = 0.22; Trial 1: P = 0.008, 95% CI [4.28, 22.71], Cohen's *d* = 1.68; Trial 2: P = 0.031, 95% CI [1.62, 24.59], Cohen's *d* = 1.42), risk assessments (trials, F(1.519, 19.75) = 14.09, P = 0.0004, ηp^2 = 0.52; group, F(1, 13) = 8.47, P = 0.012, ηp^2 = 0.39; trial × group interaction, F(2, 26) = 6.33, P = 0.005, ηp^2 = 0.32; Trial 1: P = 0.022, 95% CI [2.12, 20.77], Cohen's *d* = 1.48), and latency to press in the goal zone (trials, F(1.17, 15.30) = 18.79, P = 0.0004, ηp^2 = 0.59; group, F(1, 13) = 13.85, P = 0.002, ηp^2 = 0.51; trial × group interaction, F(2, 26) = 5.25, P = 0.01, ηp^2 = 0.28; Trial 1: P = 0.0013, 95% CI [37.71, 121.2], Cohen's *d* = 2.14) across individual conflict trials.

(**D**) aNAcC inactivation did not change the time spent in any of the evaluated zones across individual non-conflict trials.

(**E**) aNAcC inactivation did not change freezing, risk assessments, speed, and latency to press across individual non-conflict trials.

Data are mean ± SEM; *P < 0.05, **P < 0.01, Bonferroni post hoc test following two-way mixed-design ANOVA or Student's t-test. The empty circles represent individual rats, and effect sizes are reported as ηp^2 and Cohen's *d*.

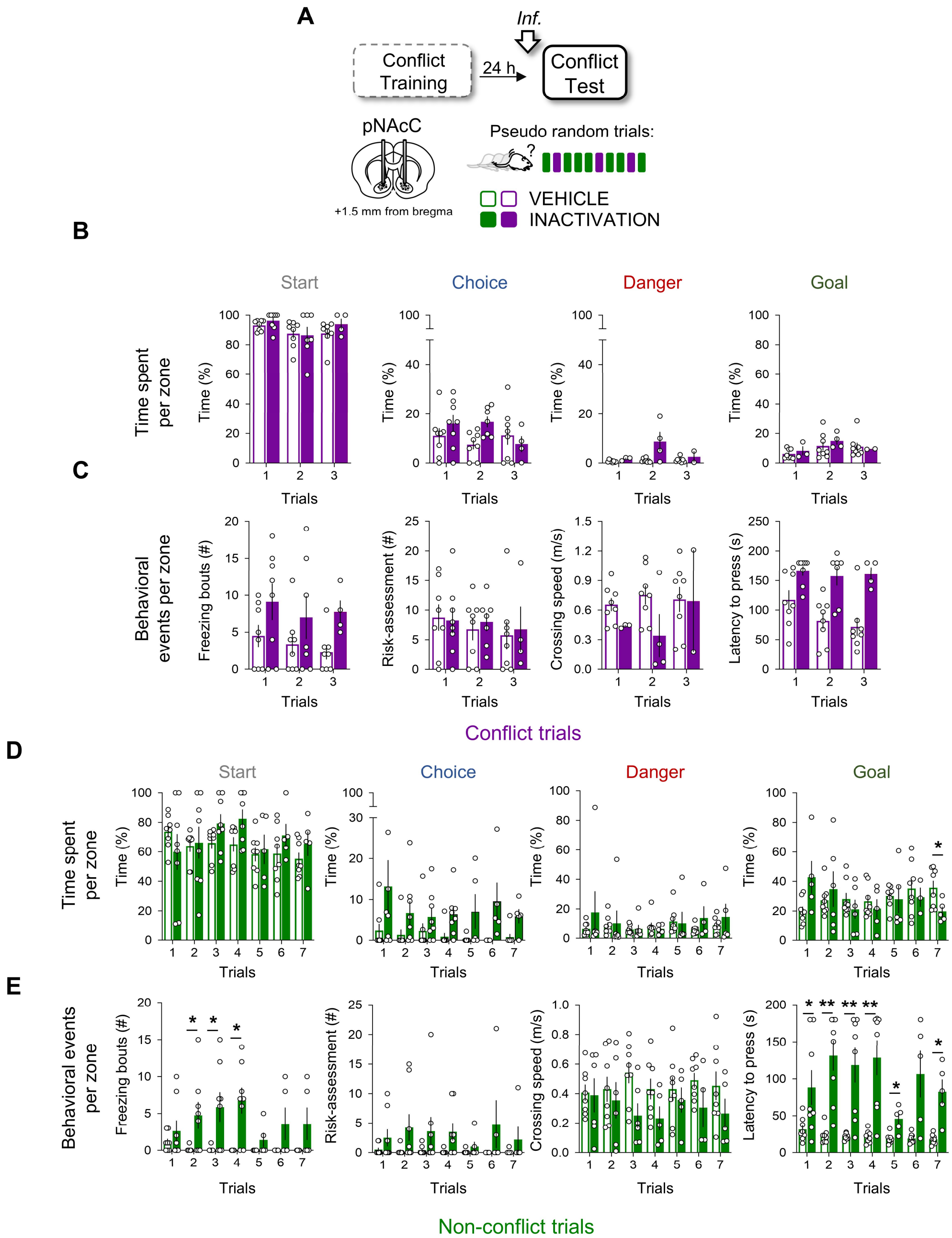

Figure S6. Trial-by-trial analysis during conflict and non-conflict behavior following pNAcC inactivation.

(**A**) Cannula placements in posterior NAcC (pNAcC). Rats received vehicle (VEH, n = 8) or muscimol+baclofen (INACT, n = 8) before the conflict test.
(**B**) pNAcC inactivation did not change the time spent in any of the evaluated zones across individual conflict trials.

(**C**) pNAcC inactivation did not alter any behavioral events evaluated or the latency to press across individual conflict trials.
(**D**) pNAcC inactivation decreased the occupancy time in the goal zone (trials, $F(3.12, 33.84) = 0.65$, $P = 0.59$, $\eta p^2 = 0.05$; group, $F(1, 12) = 0.007$, $P = 0.93$, $\eta p^2 = 0.0006$; trial × group interaction, $F(6, 65) = 2.38$, $P = 0.038$, $\eta p^2 = 0.18$). Trial 7: $P = 0.01$, 95% CI [3.61, 28.46], Cohen's $d = 1.62$) during the last non-conflict trial presentation, without affecting the time spent in other zones evaluated.
(**E**) pNAcC inactivation decreased freezing behavior (trials, $F(2.28, 27.85) = 1.25$, $P = 0.30$, $\eta p^2 = 0.09$; group, $F(1, 14) = 10.28$, $P = 0.006$, $\eta p^2 = 0.42$; trial × group interaction, $F(6, 75) = 2.64$, $P = 0.022$, $\eta p^2 = 0.17$; Trial 2: $P = 0.039$, 95% CI [-8.93, -0.31], Cohen's $d = 1.26$; Trial 3: $P = 0.024$, 95% CI [-10.51, -0.98], Cohen's $d = 1.42$; Trial 4: $P = 0.006$, 95% CI [-10.91, -2.58], Cohen's $d = 1.91$), and latency to press behavior (trials, $F(2.36, 29.58) = 2.82$, $P = 0.067$, $\eta p^2 = 0.18$; group, $F(1, 14) = 23.31$, $P = 0.0003$, $\eta p^2 = 0.62$; trial × group interaction, $F(6, 75) = 3.58$, $P = 0.0035$, $\eta p^2 = 0.22$; Trial 1: $P = 0.046$, 95% CI [-112, -1.16], Cohen's $d = 1.8$; Trial 2: $P = 0.001$, 95% CI [-156.9, -60.03], Cohen's $d = 2.61$; Trial 3: $P = 0.0046$, 95% CI [-153.3, -41.06], Cohen's $d = 2.04$; Trial 4: $P = 0.0025$, 95% CI [-163.0, -51.43], Cohen's $d = 2.25$; Trial 5: $P = 0.02$, 95% CI [-48.25, -6.42], Cohen's $d = 1.94$; Trial 7: $P = 0.01$, 95% CI [-110.6, -18.64], Cohen's $d = 2.19$) in the goal zone across individual non-conflict trials.
Data are mean ± SEM; $^*P < 0.05$, $^{**}P < 0.01$, Bonferroni post hoc test following two-way mixed-design ANOVA or Student's t-test. The empty circles represent individual rats, and effect sizes are reported as ηp^2 and Cohen's *d*.

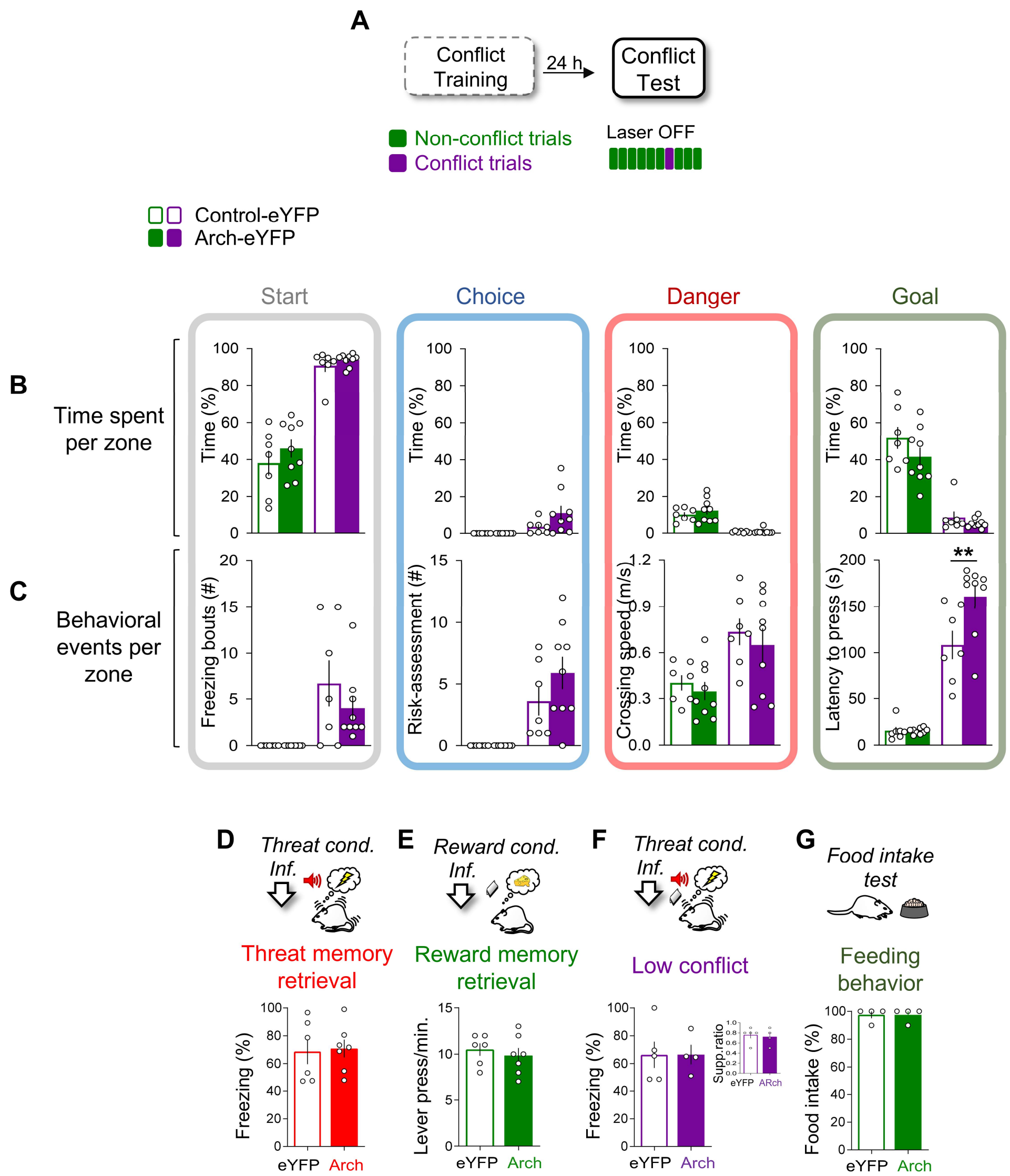

Fig. S7. Conflict test trials without optogenetic stimulation (laser off).

(**A**) After the laser ON block, rats completed a subsequent block of laser-OFF trials during the conflict test (Control-eYFP, n = 7; Arch-eYFP, n = 9).
(**B**) During laser-OFF trials, Arch-expressing rats spent similar times in the start (trials, $F(1, 14) = 144.8$, $P < 0.0001$, $\eta p^2 = 0.91$; group, $F(1, 14) = 1.48$, $P = 0.24$, $\eta p^2 = 0.09$; trial × group interaction, $F(1, 14) = 0.33$, $P = 0.57$, $\eta p^2 = 0.023$), choice (trials, $F(1, 14) = 9.61$, $P = 0.007$, $\eta p^2 = 0.40$; group, $F(1, 14) = 2.63$, $P = 0.12$, $\eta p^2 = 0.15$; trial × group interaction, $F(1, 14) = 2.62$, $P = 0.12$, $\eta p^2 = 0.15$), danger (trials, $F(1, 14) = 71.39$, $P < 0.0001$, $\eta p^2 = 0.83$; group, $F(1, 14) = 0.61$, $P = 0.44$, $\eta p^2 = 0.04$; trial × group interaction, $F(1, 14) = 0.69$, $P = 0.41$, $\eta p^2 = 0.04$) and goal zones (trials, $F(1, 14) = 90.23$, $P < 0.0001$, $\eta p^2 = 0.86$; group, $F(1, 14) = 2.76$, $P = 0.11$, $\eta p^2 = 0.16$; trial × group interaction, $F(1, 14) = 0.69$, $P = 0.41$, $\eta p^2 = 0.04$) as controls.
(**C**) Arch rats showed longer crossing latencies (trials, $F(1, 14) = 152.4$, $P < 0.0001$, $\eta p^2 = 0.91$; group, $F(1, 14) = 6.18$, $P = 0.02$, $\eta p^2 = 0.30$; trial × group interaction, $F(1, 14) = 7.39$, $P = 0.01$,

$\eta p^2 = 0.34$; Conflict trials: $P = 0.002$, 95% CI [-85.40, -18.47], Cohen's $d = 1.85$) but no differences in freezing (trials, $F(1, 14) = 16.86$, $P = 0.001$, $\eta p^2 = 0.54$; group, $F(1, 14) = 1.08$, $P = 0.315$, $\eta p^2 = 0.07$; trial × group interaction, $F(1, 14) = 1.08$, $P = 0.315$, $\eta p^2 = 0.07$), risk assessment (trials, $F(1, 14) = 27.62$, $P = 0.0001$, $\eta p^2 = 0.66$; group, $F(1, 14) = 1.65$, $P = 0.21$, $\eta p^2 = 0.10$; trial × group interaction, $F(1, 14) = 1.65$, $P = 0.21$, $\eta p^2 = 0.10$), or crossing speed (trials, $F(1, 14) = 27.70$, $P = 0.0001$, $\eta p^2 = 0.66$; group, $F(1, 14) = 0.48$, $P = 0.49$, $\eta p^2 = 0.03$; trial × group interaction, $F(1, 14) = 0.06$, $P = 0.79$, $\eta p^2 = 0.004$).

(**D–E**) No group differences were found in freezing ($t(11) = 0.19$, $P = 0.84$) or lever pressing ($t(11) = 0.60$, $P = 0.55$) during threat (Control-eYFP, n = 6; Arch-eYFP, n = 7) or reward (Control-eYFP, n = 6; Arch-eYFP, n = 7) memory retrieval tests.

(**F**) In the low-conflict test without stimulation (Control-eYFP, n = 5; Arch-eYFP, n = 4), freezing ($t(7) = 0.009$, $P = 0.99$) and suppression ratio ($t(7) = 0.32$, $P = 0.75$) were similar between groups.

(**G**) In the free food intake test without stimulation (Control-eYFP, n = 4; Arch-eYFP, n = 4), food intake was comparable ($t(6) = 0.00$, $P > 0.99$).

Zone definitions are described in Methods. Data are mean ± SEM; $^{**}P < 0.01$, Bonferroni post hoc test following two-way mixed-design ANOVA or Student's t-test. The empty circles represent individual rats, and effect sizes are reported as ηp^2 and Cohen's *d*.

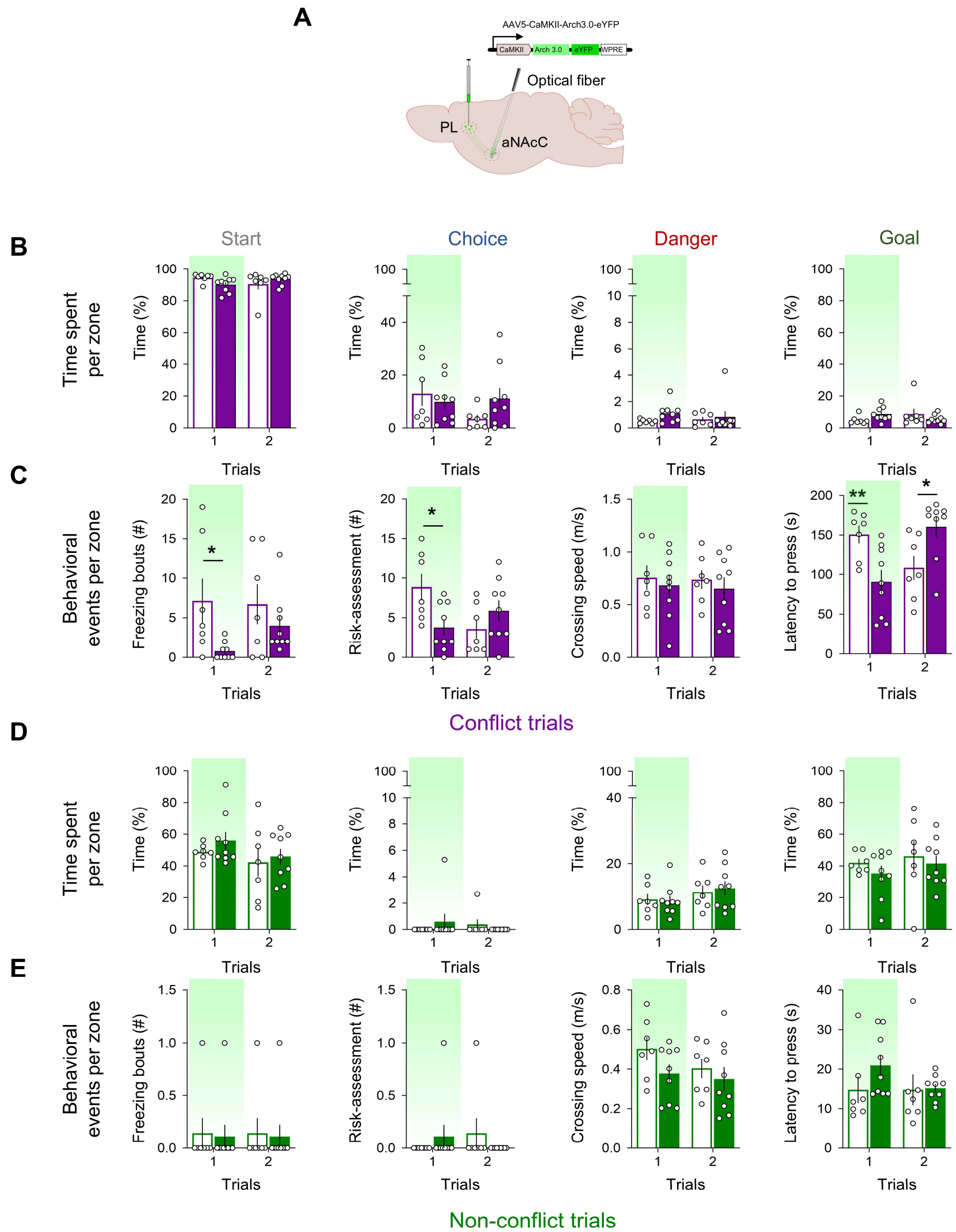

Figure S8. Trial-by-trial analysis of conflict and non-conflict behavior during PL→aNAcC photoinhibition.

(**A**) Schematic representation of viral infection in the PL and bilateral optical fiber implantation in the aNAcC.
(**B**) Time spent in the start (trials, $F(1, 14) = 0.03$, $P = 0.84$, $\eta p^2 = 0.002$; group, $F(1, 14) = 0.009$, $P = 0.76$, $\eta p^2 = 0.006$; trial × group interaction, $F(1, 14) = 6.21$, $P = 0.025$, $\eta p^2 = 0.30$; Trial 1, laser-on: $P = 0.21$, Trial 2, laser-off: $P = 0.50$), choice (trials, $F(1, 14) = 3.31$, $P = 0.09$, $\eta p^2 = 0.19$; group, $F(1, 14) = 0.27$, $P = 0.60$, $\eta p^2 = 0.01$; trial × group interaction, $F(1, 14) = 5.70$, $P = 0.03$, $\eta p^2 = 0.28$; Trial 1, laser-on: $P > 0.99$, Trial 2, laser-off: $P = 0.25$), and goal zones (trials, $F(1, 14) = 0.06$, $P = 0.79$, $\eta p^2 = 0.004$; group, $F(1, 14) = 0.01$, $P = 0.89$, $\eta p^2 = 0.001$; trial × group interaction, $F(1, 14) = 6.69$, $P = 0.025$, $\eta p^2 = 0.32$; Trial 1, laser-on: $P = 0.27$, Trial 2, laser-off: P

= 0.40) did not change between stimulated (laser-on) and unstimulated (laser off) conflict trials despite a significant interaction effect.

(**C**) Defensive behaviors, including freezing (trials, $F(1, 14) = 3.18$, $P = 0.09$, $\eta p^2 = 0.18$; group, $F(1, 14) = 3.53$, $P = 0.080$, $\eta p^2 = 0.20$; trial × group interaction, $F(1, 14) = 5.43$, $P = 0.035$, $\eta p^2 = 0.27$; Trial 1, laser-on: $P = 0.036$, 95% CI [0.35, 12.37], Cohen's $d = 1.26$), and risk assessments (trials, $F(1, 14) = 3.38$, $P = 0.08$, $\eta p^2 = 0.19$; group, $F(1, 14) = 0.74$, $P = 0.40$, $\eta p^2 = 0.05$; trial × group interaction, $F(1, 14) = 18.36$, $P = 0.0008$, $\eta p^2 = 0.56$; Trial 1, laser-on: $P = 0.018$, 95% CI [0.769, 9.38], Cohen's $d = 1.40$) decreased selectively during laser-on conflict trials but not during laser-off conflict trials. Latency to press in the goal zone (trials, $F(1, 14) = 1.08$, $P = 0.31$, $\eta p^2 = 0.07$; group, $F(1, 14) = 0.073$, $P = 0.079$, $\eta p^2 = 0.005$; trial × group interaction, $F(1, 14) = 18.33$, $P = 0.0008$ $\eta p^2 = 0.56$; Trial 1, laser-on: $P = 0.009$, 95% CI [13.42, 106.5], Cohen's $d = 1.53$; Trial 2, laser-off: $P = 0.026$, 95% CI [-98.5, -5.46], Cohen's $d = 1.33$) decreased during laser-on conflict trials and increased during laser-off conflict trials.

(**D**) Time spent in all evaluated zones did not significantly change between stimulated (laser-on) and unstimulated (laser-off) non-conflict trials.

(**E**) Behavioral events per zone and crossing latency to press were unaffected during laser-on and laser-off non-conflict trials.

Data are mean ± SEM; * $P < 0.05$, ** $P < 0.01$, Bonferroni post hoc test following two-way mixed-design ANOVA or Student's t-test. The empty circles represent individual rats, and effect sizes are reported as ηp^2 and Cohen's *d*.

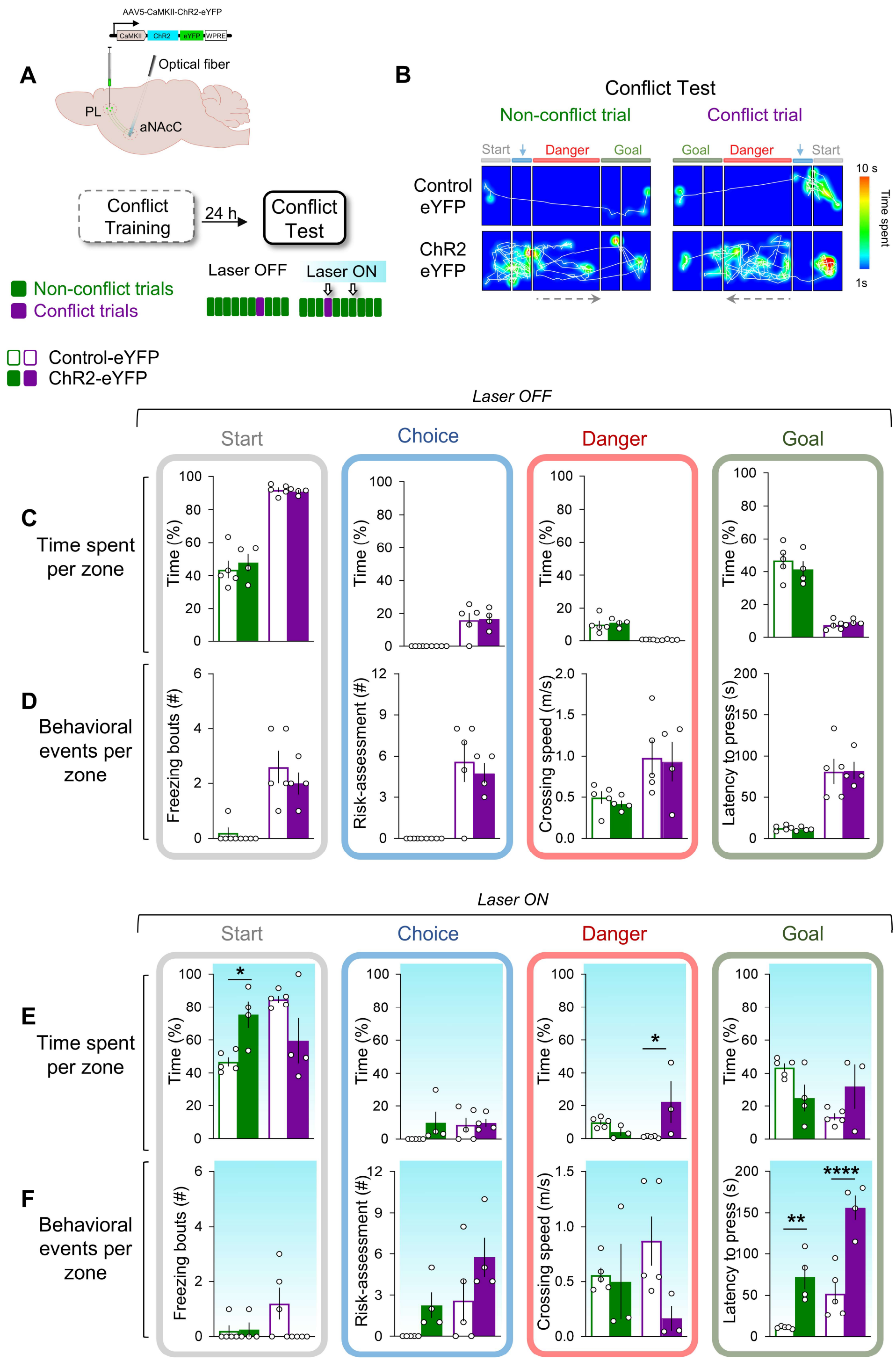

Fig. S9. Activating PL→aNAcC projection disrupts goal-directed behavior regardless of threat.

(A) Schematic representation of AAV5-CaMKII-ChR2-EYFP injection in the PL and bilateral optical fiber implantation in the aNAcC. During the conflict test (Control-eYFP, $n = 5$; ChR2-eYFP, $n = 4$), rats completed two trial blocks: laser OFF followed by laser ON.
(**B**) Representative movement tracking of eYFP and ChR2 rats during conflict and non-conflict trials.
(**C**) In laser-OFF trials, ChR2 rats spent a similar time in the start (trials, $F(1, 7) = 138.8$, $P < 0.0001$, $\eta p^2 = 0.95$; group, $F(1, 7) = 0.16$, $P = 0.70$, $\eta p^2 = 0.02$; trial × group interaction, $F(1, 7) = 0.45$, $P = 0.52$, $\eta p^2 = 0.06$), choice (trials, $F(1, 7) = 32.10$, $P = 0.0008$, $\eta p^2 = 0.82$; group, $F(1, 7) = 0.019$, $P = 0.89$, $\eta p^2 = 0.002$; trial × group interaction, $F(1, 7) = 0.019$, $P = 0.89$, $\eta p^2 = 0.002$), danger (trials, $F(1, 7) = 43.37$, $P = 0.0003$, $\eta p^2 = 0.86$; group, $F(1, 7) = 0.15$, $P = 0.70$, $\eta p^2 = 0.02$; trial × group interaction, $F(1, 7) = 0.14$, $P = 0.71$, $\eta p^2 = 0.02$), and goal zones (trials, $F(1, 7) = 117.1$, $P < 0.0001$, $\eta p^2 = 0.94$; group, $F(1, 7) = 0.24$, $P = 0.63$, $\eta p^2 = 0.03$; trial × group interaction, $F(1, 7) = 1.13$, $P = 0.32$, $\eta p^2 = 0.13$) compared to controls.
(**D**) No differences were observed in freezing in the start zone (trials, $F(1, 7) = 26.89$, $P = 0.0013$, $\eta p^2 = 0.79$; group, $F(1, 7) = 1.13$, $P = 0.32$, $\eta p^2 = 0.13$; trial × group interaction, $F(1, 7) = 0.22$, $P = 0.65$, $\eta p^2 = 0.03$), risk assessment (trials, $F(1, 7) = 32.08$, $P = 0.0008$, $\eta p^2 = 0.82$; group, $F(1, 7) = 0.21$, $P = 0.65$, $\eta p^2 = 0.03$; trial × group interaction, $F(1, 7) = 0.21$, $P = 0.65$, $\eta p^2 = 0.03$), crossing speed (trials, $F(1, 7) = 7.77$, $P = 0.02$, $\eta p^2 = 0.52$; group, $F(1, 7) = 0.16$, $P = 0.69$, $\eta p^2 = 0.02$; trial × group interaction, $F(1, 7) = 0.006$, $P = 0.93$, $\eta p^2 = 0.0009$), or press latency (trials, $F(1, 7) = 50.42$, $P = 0.0002$, $\eta p^2 = 0.87$; group, $F(1, 7) = 0.001$, $P = 0.97$, $\eta p^2 = 0.0002$; trial × group interaction, $F(1, 7) = 0.01$, $P = 0.90$, $\eta p^2 = 0.002$).
(**E**) Photoactivation increased start-zone occupancy during non-conflict trials (trials, $F(1, 7) = 2.09$, $P = 0.19$, $\eta p^2 = 0.22$; group, $F(1, 7) = 0.05$, $P = 0.81$, $\eta p^2 = 0.008$; trial × group interaction, $F(1, 7) = 12.08$, $P = 0.01$, $\eta p^2 = 0.63$; Non-conflict trials: $P = 0.03$, 95% CI [-54.43, -2.65], Cohen's $d = 1.85$) and danger-zone occupancy during conflict trials (trials, $F(1, 6) = 1.38$, $P = 0.28$, $\eta p^2 = 0.18$; group, $F(1, 6) = 1.94$, $P = 0.21$, $\eta p^2 = 0.24$; trial × group interaction, $F(1, 6) = 10.69$, $P = 0.01$, $\eta p^2 = 0.64$; Conflict trials: $P = 0.018$, 95% CI [-38.54, -3.65], Cohen's $d = 2.26$).
(**F**) Photoactivation significantly prolonged press latency in both trial types (trials, $F(1, 7) = 53.51$, $P = 0.0002$, $\eta p^2 = 0.88$; group, $F(1, 7) = 32.97$, $P = 0.0007$, $\eta p^2 = 0.82$; trial × group interaction, $F(1, 7) = 6.26$, $P = 0.040$, $\eta p^2 = 0.47$; Non-conflict trials: $P = 0.005$, 95% CI [-103.3, -19.22], Cohen's $d = 2.45$; Conflict trials: $P < 0.0001$, 95% CI [-146.1, -62.07], Cohen's $d = 4.16$), but did not affect freezing (trials, $F(1, 7) = 1.29$, $P = 0.29$, $\eta p^2 = 0.15$; group, $F(1, 7) = 2.068$, $P = 0.19$, $\eta p^2 = 0.22$; trial × group interaction, $F(1, 7) = 3.60$, $P = 0.09$, $\eta p^2 = 0.33$), risk assessment (trials, $F(1, 7) = 11.09$, $P = 0.01$, $\eta p^2 = 0.61$; group, $F(1, 7) = 4.00$, $P = 0.08$, $\eta p^2 = 0.36$; trial × group interaction, $F(1, 7) = 0.24$, $P = 0.63$, $\eta p^2 = 0.03$), or crossing speed (trials, $F(1, 6) = 0.002$, $P = 0.96$, $\eta p^2 = 0.0004$; group, $F(1, 6) = 4.46$, $P = 0.07$, $\eta p^2 = 0.42$; trial × group interaction, $F(1, 6) = 2.03$, $P = 0.20$, $\eta p^2 = 0.25$).
Zone definitions are described in Methods. Data are mean ± SEM; *$P < 0.05$, **$P < 0.01$, ****$P < 0.0001$, Bonferroni post hoc tests following two-way mixed-design ANOVA. The empty circles represent individual rats, and effect sizes are reported as ηp^2 and Cohen's *d*.

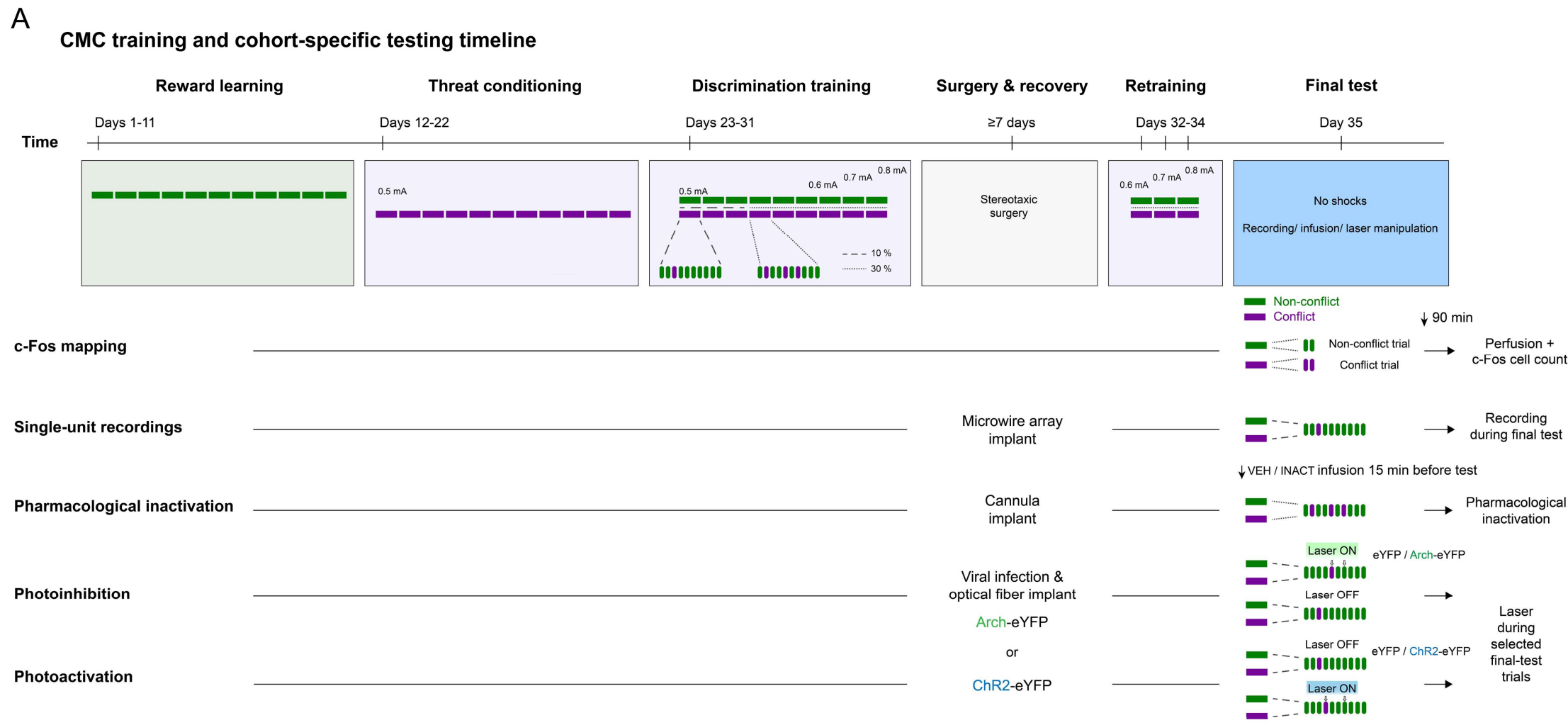

Fig. S10. Experimental design across CMC cohorts.

Schematic overview of the behavioral training and final testing procedures used across experimental cohorts. All rats underwent the same core CMC behavioral sequence, including reward learning, threat conditioning, discrimination training, and a final memory-guided conflict test. Surgical procedures, post-surgical retraining, recordings, pharmacological inactivation, optogenetic manipulation, and c-Fos mapping were introduced according to the requirements of each experimental cohort. The final conflict test was conducted without shock delivery, allowing behavior to be interpreted as memory-guided integration of learned reward and threat cues rather than as a response to immediate shock.

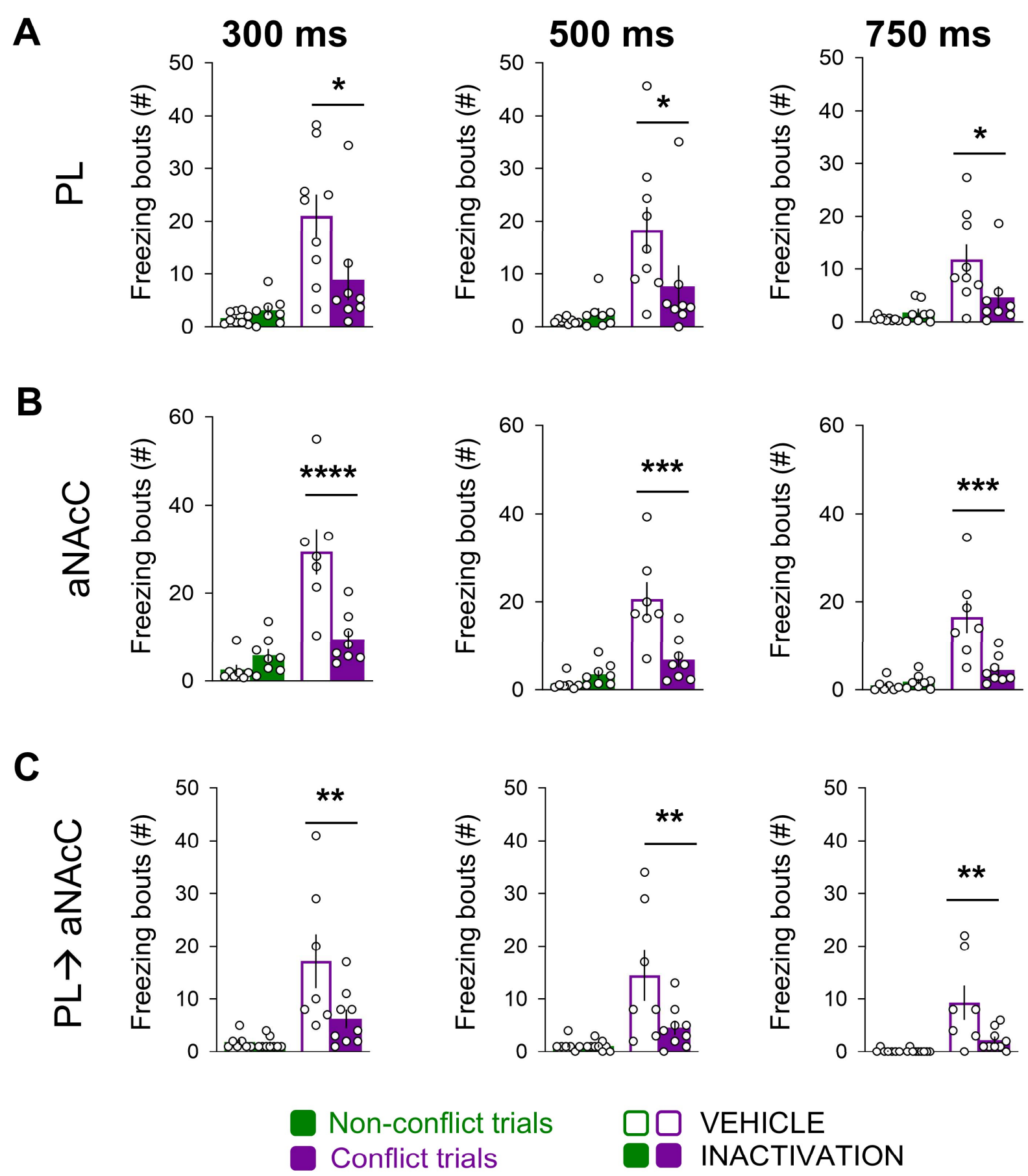

Figure S11. Freezing behavior at different time thresholds following inactivation and photoinhibition manipulations.

(**A**) PL inactivation decreased freezing behavior in conflict trials during 300 ms (trials, $F(1, 15) = 23.40$, $P = 0.0002$, $\eta p^2 = 0.60$; group, $F(1, 15) = 3.02$, $P = 0.10$, $\eta p^2 = 0.16$; trial × group interaction, $F(1, 15) = 6.82$, $P = 0.019$, $\eta p^2 = 0.31$; Conflict trials: $P = 0.010$, 95% CI [2.63, 21.62], Cohen's $d = 1.46$), 500 ms (trials, $F(1, 15) = 16.27$, $P = 0.001$, $\eta p^2 = 0.52$; group, $F(1, 15) = 2.07$, $P = 0.17$, $\eta p^2 = 0.11$; trial × group interaction, $F(1, 15) = 4.78$, $P = 0.04$, $\eta p^2 = 0.24$; Conflict trials: $P = 0.037$, 95% CI [0.55, 20.87], Cohen's $d = 1.20$), and 750 ms (trials, $F(1, 15) = 16.33$, $P = 0.001$, $\eta p^2 = 0.52$; group, $F(1, 15) = 2.47$, $P = 0.13$, $\eta p^2 = 0.14$; trial × group interaction, $F(1, 15) = 5.87$, $P = 0.02$, $\eta p^2 = 0.248$; Conflict trials: $P = 0.017$, 95% CI [1.12, 13.27], Cohen's $d = 1.35$).
(**B**) aNAcC inactivation decreased freezing behavior in conflict trials during 300 ms (trials, $F(1, 13) = 52.24$, $P < 0.0001$, $\eta p^2 = 0.80$; group, $F(1, 13) = 6.28$, $P = 0.026$, $\eta p^2 = 0.3$; trial × group interaction, $F(1, 13) = 30.19$, $P = 0.0001$, $\eta p^2 = 0.69$; Conflict trials: $P < 0.0001$, 95% CI [10.56, 29.29], Cohen's $d = 2.61$), 500 ms (trials, $F(1, 13) = 43.26$, $P < 0.0001$, $\eta p^2 = 0.76$; group, $F(1 ,13) = 5.96$, $P = 0.029$, $\eta p^2 = 0.31$; trial × group interaction, $F(1, 13) = 21.78$, $P = 0.0004$, $\eta p^2 =$

0.62; Conflict trials: $P = 0.0002$, 95% CI [6.83, 20.91], Cohen's $d = 2.42$), and 750 ms (trials, $F(1, 13) = 32.62$, $P < 0.0001$, $\eta p^2 = 0.71$; group, $F(1, 13) = 7.23$, $P = 0.018$, $\eta p^2 = 0.35$; trial × group interaction, $F(1, 13) = 16.37$, $P = 0.001$, $\eta p^2 = 0.55$; Conflict trials: $P = 0.0002$, 95% CI [5.82, 18.32], Cohen's $d = 2.37$).

(**C**) PL→ aNAcC photoinhibition decreased freezing behavior in conflict trials during 300 ms (trials, $F(1, 14) = 20.99$, $P = 0.0004$, $\eta p^2 = 0.59$; group, $F(1, 14) = 4.19$, $P = 0.059$, $\eta p^2 = 0.23$; trial × group interaction, $F(1, 14) = 5.94$, $P = 0.028$, $\eta p^2 = 0.29$; Conflict trials: $P = 0.008$, 95% CI [2.63, 19.21], Cohen's $d = 1.57$), 500 ms (trials, $F(1, 14) = 15.81$, $P = 0.0014$, $\eta p^2 = 0.5$; group, $F(1, 14) = 4.39$, $P = 0.054$, $\eta p^2 = 0.23$; trial × group interaction, $F(1, 14) = 5.40$, $P = 0.035$, $\eta p^2 = 0.27$; Conflict trials: $P = 0.008$, 95% CI [2.35, 17.40], Cohen's $d = 1.56$), and 750 ms (trials, $F(1, 14) = 14.37$, $P = 0.002$, $\eta p^2 = 0.50$; group, $F(1, 14) = 6.14$, $P = 0.026$, $\eta p^2 = 0.30$; trial × group interaction, $F(1, 14) = 5.61$, $P = 0.03$, $\eta p^2 = 0.28$; Conflict trials: $P = 0.003$, 95% CI [2.18, 11.95], Cohen's $d = 1.72$).

Data are mean ± SEM; *$P< 0.05$, **$P < 0.01$, ***$P < 0.001$, ****$P < 0.0001$, Bonferroni post hoc test following one-way mixed-design ANOVA or Student's t-test. The empty circles represent individual rats, and effect sizes are reported as ηp^2 and Cohen's *d*.

Movie S1. Representative behavior during conflict and non-conflict trials in the conflict test.

Automated video tracking of a rat performing the crossing-mediated conflict task during the conflict test (no shocks delivered), shown at 2× speed. The video depicts successive trials in which the animal decides whether to cross an electrified grid to obtain food. In conflict trials, the reward cue (reward light ON) is presented together with the danger cue (white noise LED ON), requiring the animal to withhold approach in the face of potential threat. In non-conflict trials, only the reward cue is presented, allowing safe reward pursuit. Trajectories and zone occupancy were recorded using automated tracking software.

Experiment	Initial n	Final analyzed n	Exclusions and reasons
Fig.1 D-E, Fig. S2	24	17 (VEH=9, INACT= 8)	2 (Failed to meet behavioral task criteria)<br>5 (cannula misplacement)
Fig. 1 F	24	20 (VEH= 9, INACT= 11)	4 (cannula misplacement)
Fig. 1 G	14	11 (VEH= 5, INACT= 6)	3 (cannula misplacement)
Fig. 1 H	28	27 (VEH= 14, INACT= 13)	1 (cannula misplacement)
Fig. 1 I	14	12 (VEH= 6, INACT= 6)	2 (cannula misplacement)
Fig . 1 J	14	10 (VEH= 5, INACT= 5)	2 (cannula misplacement)<br>2 (injector blockage)
Fig. 2 B-G	8	5	2 (absence of detectable single units)<br>1 (health issues after electrode implant)
Fig. 2 H-J	7	7 (Conflict= 4, non-conflict= 3)	0
Fig. 3 C-D, Fig. S5	22	15 (VEH= 7, INACT= 8)	2 (Failed to meet behavioral task criteria)<br>4 (cannula misplacement)<br>1 (implant detachment)
Fig. 3 E	14	11 (VEH= 5, INACT= 6)	3 (cannula misplacement)
Fig. 3 F	14	11 (VEH= 6, INACT= 5)	3 (cannula misplacement)
Fig. 3 G	14	11 (VEH= 5, INACT= 6)	3 (cannula misplacement)
Fig. 3 H	14	10 (VEH= 5, INACT= 5)	4 (cannula misplacement)
Fig. 3 I	14	11 (VEH= 6, INACT= 5)	3 (cannula misplacement)
Fig. 4 C-D, Fig. S6	22	16 (VEH= 8, INACT= 8)	2 (Failed to meet behavioral task criteria)<br>3 (cannula misplacement)<br>1 (implant detachment)
Fig. 4 E	14	11 (VEH= 6, INACT= 5)	3 (cannula misplacement)
Fig. 4 F	14	10 (VEH= 5, INACT= 5)	2 (cannula misplacement)<br>2 (injector blockage)
Fig. 4 G	14	11 (VEH= 5, INACT= 6)	3 (cannula misplacement)
Fig. 4 H	14	12 (VEH= 6, INACT= 6)	2 (cannula misplacement)
Fig. 5 A	8	5	3 (optical fiber and/or viral injection misplacement)
Fig. 5 C-F, Fig. S7	24*	16 (eYFP= 7, Arch= 9)	1 (Failed to meet behavioral task criteria), 5 (optical fiber and/or viral injection misplacement), 2 (implant detachment)

Fig. 5 G-H	24*	13 (eYFP= 6, Arch= 7)	1 (Failed to meet behavioral task criteria), 6 (optical fiber and/or viral injection misplacement), 4 (implant detachment)
Fig. 5 I	14*	9 (eYFP= 5, Arch= 4)	4 (optical fiber and/or viral injection misplacement), 1 (implant detachment)
Fig. 5 J	14*	7 (eYFP= 3, Arch= 4)	4 (optical fiber and/or viral injection misplacement), 3 (implant detachment)
Fig. 5 K	14*	8 (eYFP= 4, Arch= 4)	4 (optical fiber and/or viral injection misplacement), 2 (implant detachment)
Fig. 6 C	28	23 Non-conflict (VEH=6, INACT= 6); Conflict (VEH= 5, INACT= 6)	1 (Failed to meet behavioral task criteria), 3 (cannula misplacement), 1 (implant detachment)
Fig. 6 D	32	28 Non-conflict (VEH=5, INACT= 5); Conflict (VEH= 9, INACT= 9)	4 (cannula misplacement)
Fig. 6 E	28	17 Non-conflict (eYFP=3, Arch= 5); Conflict (eYFP= 4, Arch= 5)	2 (Failed to meet behavioral task criteria), 6 (optical fiber and/or viral injection misplacement), 2 (health issues after implant), 1(implant detachment)
Fig. S1 A-D	16	11 (VEH= 6, INACT= 5)	5 (cannula misplacement)
Fig. S9 A-F	14	9 (eYFP= 5, ChR2= 4)	3 (optical fiber and/or viral injection misplacement), 2 (implant detachment)

Table S1. Summary of sample sizes and exclusion criteria. Initial number of animals, final number included in each experiment, and reasons for exclusion. *The same cohort of animals was used across these experiments.
